# A scoping review of the evidence on survivorship care plans among minority, rural, and low-income populations

**DOI:** 10.1007/s11764-024-01609-z

**Published:** 2024-06-22

**Authors:** Willi L. Tarver, Zion Justice, Pallavi Jonnalagadda, Saurabh Rahurkar, Samilia Obeng-Gyasi, Jessica L. Krok-Schoen, Abigail Petrecca, Electra D. Paskett

**Affiliations:** 1 James Comprehensive Cancer Center, The Ohio State University, Columbus, OH USA; 2 Division of Cancer Prevention and Control, Department of Internal Medicine, College of Medicine, The Ohio State University, Columbus, OH USA; 3 The Center for the Advancement of Team Science, Analytics, and Systems Thinking in Health Services and Implementation Science Research (CATALYST), College of Medicine, The Ohio State University, Columbus, OH USA; 4 Wilberforce University, Wilberforce, OH USA; 5 Department of Biomedical Informatics, College of Medicine, The Ohio State University, Columbus, OH USA; 6 Division of Surgical Oncology, Department of Surgery, The Ohio State University, Columbus, OH USA; 7 Division of Health Sciences, School of Health and Rehabilitation Sciences, The Ohio State University, Columbus, OH USA

**Keywords:** Cancer, Survivorship care plans, Cancer survivorship, Systematic review, Underserved populations

## Abstract

**Purpose:**

Despite recent advances in cancer control and the number of cancer survivors increasing substantially over the past years, some cancer survivors continue to experience disparities due to barriers to recommended survivorship care. The use of survivorship care plans (SCPs) may be a way to help care for these individuals and their respective issues after they complete their primary treatment. The purpose of this scoping review is to understand the evidence on SCPs among minority, rural, and low-income populations: groups that experience disproportionately poorer cancer health outcomes.

**Methods:**

Computer-based searches were conducted in four academic databases. We included peer-reviewed studies published in the English language and conducted in the USA. We systematically extracted information from each paper meeting our inclusion criteria.

**Results:**

Our search identified 45 articles. The 4 major themes identified were (1) disparities in the receipt of SCPs where populations experience unmet needs; (2) benefits of SCPs, including improved care coordination and self-management of cancer; (3) needs and preferences for survivorship care; and (4) barriers and facilitators to using SCPs.

**Conclusions:**

Despite the potential benefits, underserved cancer survivors experience disparities in the receipt of SCPs and continue to have unmet needs in their survivorship care. Survivorship care may benefit from a risk-stratified approach where SCPs are prioritized to survivors belonging to high-risk groups.

**Implications for Cancer Survivors:**

SCPs are a tool to deliver quality care for cancer survivors. While evidence is mixed on SCPs’ benefits among the general population, SCPs show promise for underserved populations when it comes to proximal outcomes that contribute to disparities.

**Supplementary Information:**

The online version contains supplementary material available at 10.1007/s11764-024-01609-z.

## Introduction

Cancer is a major public health problem in the USA with more than 1.9 million new cases of cancer expected to be diagnosed in 2023 [1]. It is estimated that approximately 18 million individuals with a history of cancer were alive in the USA in January 2022 [2]. Importantly, this number is expected to increase to more than 22.1 million individuals by January 2030 [3]. Due to recent advancements in early detection and treatment, many adult cancer survivors can expect to live for decades; however, optimal cancer care is not universally available and can result in disparities for medically underserved populations [4, 5]. Medically underserved populations are described as groups of people with cultural or economic barriers hindering them from receiving adequate medical care services [6]. These groups can include different racial and ethnic minorities, those with lower socioeconomic status (SES), and those living in rural geographic areas who have disproportionate cancer burdens when compared to people who have more access to better quality of care [7]. Populations that experience barriers to receiving high-quality cancer treatment may also experience disparities in survivorship care and survivorship experiences [8, 9]. This creates the need for effective survivorship care among these vulnerable populations of cancer survivors to manage the impact of their cancer treatment.

The Institute of Medicine (IOM) highlighted the growing concerns surrounding the lack of clarity of what to expect after treatment by both patients and providers [10]. There are many health effects that are associated with a cancer diagnosis [2, 11] such as mental health issues, long-term or late effects, stress, and the recurrence of cancers [12–14]. Importantly, survivorship care following treatment should be patient-centered and tailored to the unique needs, experiences, and challenges of each patient [15]. One way to help care for these individuals and their respective issues is the use of survivorship care plans (SCPs) [10]. These detailed plans provided to patients after they complete their primary treatment contain a summary of their cancer treatment and recommendations for follow-up care. The IOM identified survivorship care planning as a key component in ensuring quality care for cancer survivors [10]. As a result, several leading organizations embraced this recommendation, including the American Society of Clinical Oncology (ASCO), a leader in creating templates which were mandated through the American College of Surgeon’s Commission on Cancer (CoC). SCPs are individualized guidelines detailing how to properly take care of, monitor, and maintain a cancer survivor’s health [11]. They include treatment summaries, upcoming surveillance/follow-up visits, other cancer screenings, and healthy behaviors to reduce the risk of cancer recurrence and/or secondary cancer. While existing research provides little evidence that SCPs improve health outcomes and healthcare delivery, findings are more positive for proximal outcomes such as information received and care delivery, particularly when SCPs are accompanied by counseling to prepare cancer survivors for future clinical encounters [16]. Furthermore, while cancer survivors show satisfaction with SCPs [17], they still experience unmet needs post-treatment which could result from resource barriers hindering their proper implementation [17].

Recent attention has been given to potential disparities in cancer survivorship outcomes among groups that are underserved. For example, rural and low-income survivors experience gaps in supportive care during and after the completion of treatment [18]. In addition, a previous review examining the use of SCPs among racial and ethnic minority female breast cancer survivors found that receiving SCPs was desired by this population but given sporadically [19]. Although studies explored the use of SCPs among other groups that are underserved such as those living in rural or remote areas or low-income populations, these studies were not systematically reviewed and synthesized to date. The objective of this study is to conduct a scoping review to understand the evidence on SCPs among various underserved populations.

## Methods

For this study, we followed the scoping review methodology proposed by Arksey and O’Malley which informed the following steps: (1) identifying the research question, (2) identifying relevant studies, (3) study selection, (4) data charting, and (5) collating and reporting results [20]. We chose this methodology given that scoping reviews are often used to map a body of literature on a topic and identify knowledge gaps [21]. We present steps 1 through 5 of our scoping review below.

### Step 1: Identifying the research question

The main research question guiding our review was: “What is the evidence on survivorship care plans among underserved populations?” Our scoping review aimed to analyze and synthesize the available evidence on SCPs among minority, rural, and low-income populations. This review will contribute to further cancer SCP development by addressing the following issues: (1) understanding the use of SCPs among groups that are underserved, (2) identifying the barriers that healthcare organizations face when trying to implement plans into their post-treatment care and tailor them to groups that are underserved, and (3) providing insights for future research directions.

### Step 2: Identifying relevant studies

#### Search strategy

Our study followed the recommendations of the statements on Enhancing Transparency in Reporting the Synthesis of Qualitative Research (ENTREQ) and the Preferred Reporting Items for Systematic Reviews and Meta-Analyses (PRISMA) guidelines [22, 23]. Web-based searches were conducted in the following academic databases: (1) PubMed (cancer subset), (2) MEDLINE, (3) PsycINFO, and (4) CINAHL. To optimize the search results, we used various combinations of keywords taken from the existing literature and Medical Subject Headings terms (see Appendix 1). Lastly, we identified additional studies using a snowball searching technique where we examined the reference lists of all included studies that met our inclusion criteria.

#### Inclusion criteria

We identified papers that appeared in peer-reviewed journals and were published in the English language from January 2005 through December 2023. We limited our search to studies published after 2005, the year the seminal IOM report, *From Cancer Patient to Cancer Survivor: Lost in Transition*, was published highlighting the need for effective survivorship care [10]. Like other reviews focused on groups that are underserved, we limited eligible articles to those published in English, from studies conducted in the USA [24]. Studies were included if they focused on SCPs in the following underserved groups: racial and ethnic minorities, low SES populations, and rural populations. Underserved populations had to make up at least 30% of study participants. Studies that did not focus on these underserved groups but conducted sub-group analyses to identify factors related to these populations and present findings specific to the included underserved groups were also eligible. For such studies, we lowered our threshold to 20% study participants representing underserved groups. Studies also had to present results on these groups separately and not as part of the overall sample. However, during full-text review, we made an exception for studies using nationally representative datasets such as BRFSS. Even though all findings were not reported separately for some studies, these studies provided important evidence on SCPs.

### Step 3: Study selection

Each study was individually assessed for relevance. Any disagreements between reviewers were reconciled by consensus. We used a two-step inclusion process to identify articles that met our inclusion criteria. In step 1, we examined paper titles and abstracts and excluded studies that clearly did not have a focus on either SCPs or cancer or groups that are underserved. Each citation was independently reviewed by all reviewers (WLT, PJ, SR, and AP). Disagreements between reviewers were reconciled by consensus. In step 2, the full-text papers of the remaining citations were obtained for independent assessment of all inclusion criteria.

### Step 4: Data charting

Data extraction was done in duplicate by WLT and PJ, independently. As in step 1, any disagreements were reconciled by consensus. We systematically extracted the following information from each of the papers included in our review: the populations of interest (African Americans, Hispanics, low-income populations, rural, etc.), the cancer type (breast cancer, pancreatic cancer, lung cancer, etc.), study design (cohort study, randomized controlled trial, etc.), details about the SCPs (paper or electronic), and whether there was any evidence of tailoring of the SCPs.

### Step 5: Collating and reporting results

Using the information from the data extraction, we created descriptive and numerical summaries of the information from published studies related to our scoping review objectives. Details related to SCP and SCP components, factors that influenced their use and effectiveness, were extracted and synthesized. Within results and discussions, we identified themes and sub-themes. We conducted a qualitative thematic analysis and grouped findings into four major themes derived from their meaning. This process was conducted through a series of discussions among the authors until a consensus was reached.

## Results

### Included studies

**Fig. 1:**
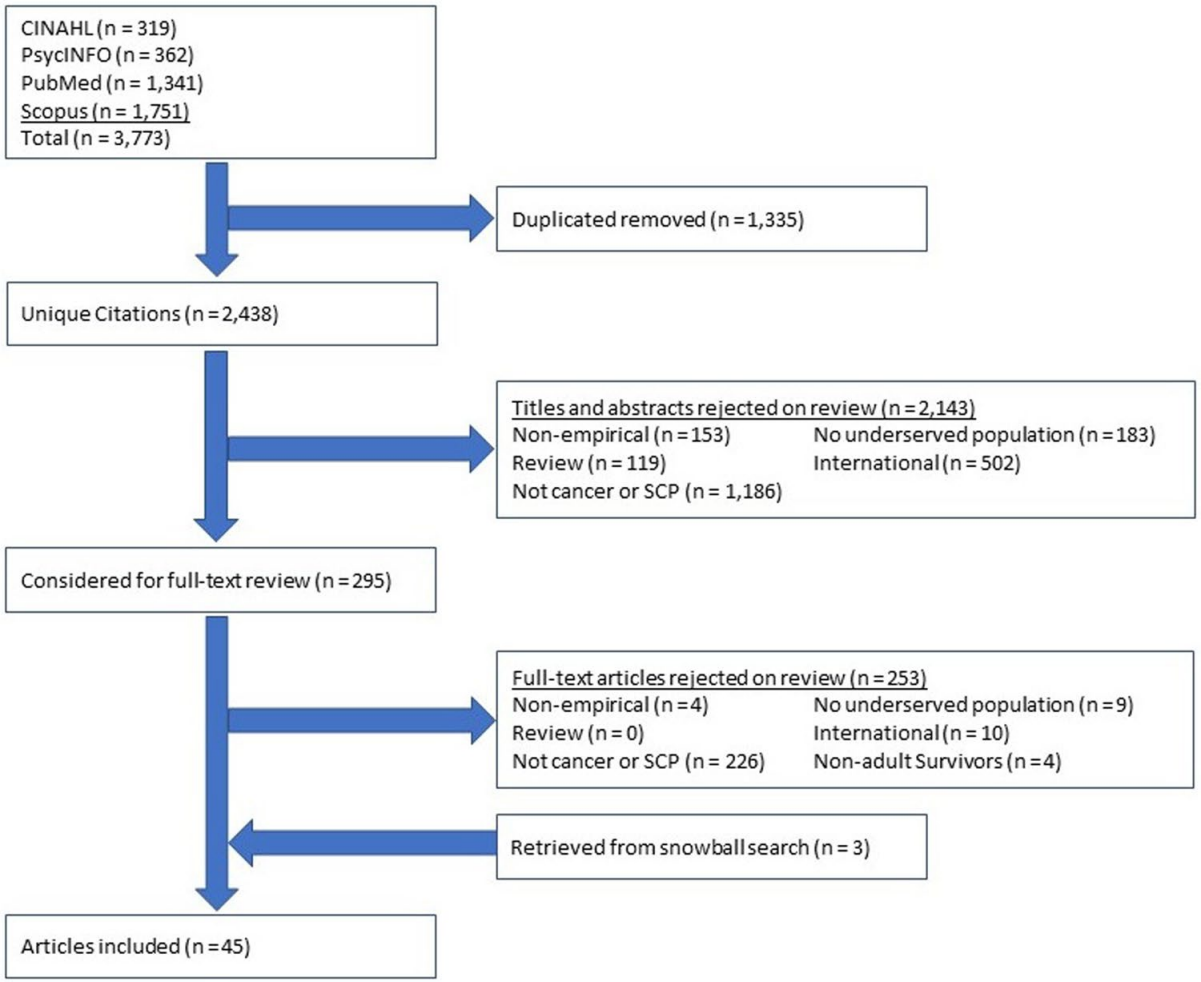
Scoping review flowchart

Our keyword search identified an initial yield of 2438 unique articles. Studies in step 1 were most frequently excluded for not having a focus on cancer or SCPs, being conducted outside of the USA, and not targeting a group that is underserved (see Fig. 1). After excluding these citations, 295 articles were included in step 2. The primary reasons for exclusion at this stage included not having a focus on cancer or SCPs and being conducted outside of the USA. After applying all the exclusion criteria in the review of full-text articles, 42 studies were included. The snowball search yielded an additional 3 articles. Data was extracted from the final set of articles. A total of 45 studies were included in this systematic review published between 2009 and 2023 (see Appendix 2 for a list of included studies).

### Study characteristics

Twenty-three of the included studies used quantitative methods, 14 used qualitative methods, and 8 used mixed methods. Most quantitative studies (16/23) were cross-sectional studies, 2 were cohort studies, 4 were randomized control trials (RCTs), and 1 was a single-arm study. Six of the 14 qualitative studies used focus group discussions, 4 used semi-structured interviews, 3 used community-based participatory research (CBPR) approaches (consensus meetings, focus groups, interviews), and 1 was a think aloud. Studies varied with respect to the population targeted, type of SCPs administered (treatment summary or follow-up care plan), and the cancer type of interest. The majority of studies focused on Black populations followed by studies targeting diverse populations of more than one group that is underserved. In addition, most studies focused on breast cancer (*n* = 29) survivors, followed by colorectal (*n* = 11), gynecologic (*n* = 11), and prostate cancer (*n* = 10) survivors.

### Theme 1: Benefits of survivorship care plans

One recurring theme we identified was related to benefits from SCPs in 18 articles (see Table 1). Eleven studies were quantitative composed of 3 RCTs, 1 single-arm trial, 6 cross-sectional studies, and 1 cohort study. Four were qualitative composed of 2 CBPR (focus groups, RAND-Delphi method, and interviews), 1 focus group study, and 1 think aloud. Two of the 3 mixed methods studies were single-arm studies, 1 was an RCT, and all 3 used semi-structured interviews. Participants including both healthcare providers and survivors across different studies found the SCP to be useful [25, 26]. Some articles studied the effects of SCP interventions that entailed some form of patient navigation through counseling with nurses or nutritionists [27–29]. For example, Maly and colleagues conducted an RCT of SCPs for a low-income, predominantly Latina population of breast cancer survivors [29]. This study examined the effects of treatment summaries and SCPs coupled with a nurse counseling session on physician implementation of survivorship care. Participants in the intervention group reported greater physician implementation of survivorship care such as treatment of hot flashes. Herschman and colleagues conducted an RCT among women with early-stage breast cancer [28]. Participants in the intervention group met with a nurse/nutritionist who provided them with a treatment summary, surveillance, and lifestyle recommendations. This study found no significant improvements in patient-reported outcomes like treatment satisfaction, survivor concerns, depression, and impact of cancer; however, the intervention was associated with decreased worry in the short term. In a separate publication, this group also found that the intervention was effective at changing behaviors and improving knowledge in the short term and was less effective among Hispanic people in improving attitudes towards healthy eating and physical activity when compared with non-Hispanic White individuals [27].

**Table 1:** Benefits from survivorship care plans among underserved populations (*n* = 18)

Authors	Underserved population	Sample and cancer type	SCP details	Outcomes	Study design	Key findings
Baseman et al. (2017)	Rural	*n* = 11 (6 survivors, 4 primary care providers (experience in rural settings), 1 oncologist) Breast	Mobile phone app (SmartSurvivor) and website • Care team • Treatment summary (diagnosis, radiation treatment summaries, etc.) • Follow-up care (e.g., ongoing toxicities to track, wellness/concerns, recommended follow-up schedule/frequency, etc.) • Tracking tool for self-monitoring	Feasibility and acceptability of SCP as mHealth app (SmartSurvivor)	Qualitative; think aloud	• One-stop shop; mHealth SCP supports coordination of care with specialists • Survivors and providers empowered by the tracking feature of SmarSurvivor • Providers said the report function saved time during a visit; the journaling feature supports improved patient-provider communication • Reminders/notifications/tips features were seen as useful for surveillance by both survivors and providers
Casillas et al. (2011)	Low educational attainment Minority	*n* = 376 Leukemia/lymphoma, bone and soft tissue sarcomas	No specific SCPs evaluated	Self-reported receipt of survivorship care planning Expectations from providers Confidence in managing survivorship care	Quantitative; cross-sectional	• Ethnic minority survivors were associated with higher odds of reporting low confidence in managing survivorship care
Casillas et al. (2021)	Latino	*n* = 103 (47 adolescents and young adults, 56 family members) Leukemia/lymphoma, brain/central nervous system, other solid tumors	Photonovela survivorship story Actionable survivorship care planning tools Sample customizable SCP	Assess the efficacy of photonovela intervention on the following: • Patient and family confidence in survivorship care management • Cancer stigma • Knowledge related to late effects and the need for consistent survivorship care	Quantitative; single-arm trial	• Increase in survivor and family member confidence in the management of survivorship care • Increase in family knowledge of late effects and issues related to survivorship care
Casillas et al. (2022)	Latino	*n* = 34 (adolescents and young adults, 16 parents and family members) Leukemia, lymphoma, brain/central nervous system, bone, soft tissue	Photonovela • Photonovela survivorship story • Actionable survivorship care planning tools • Sample customizable SCP	Develop and test the acceptability of a culturally appropriate photonovela intervention to improve knowledge of, and the need to receive, survivorship care	Qualitative; community-partnered participatory research (RAND-Delphi method, focus groups)	• SCP helps navigate the system • SCP is helpful for providers not familiar with cancer history • Survivors are comfortable meeting the providers alone without family if SCP available • All participants received the photonovela positively. They liked the peer explanation of an SCP
Duggan et al. (2023)	Rural	*n* = 72 Breast, prostate, colorectal, lymphoma	SCP with coaching from a lay health educator (intervention), SCP alone (control)	Health literacy Self-efficacy Quality of life	Mixed; RCT; semi-structured interviews	• No change in accuracy or participant knowledge • Significant change in knowledge about the type of surgery received in the control group • No change in self-efficacy and health-related quality of life, significant change in self-efficacy among those in the intervention arm with marginal health literacy at baseline • 22% of participants discussed SCP with both PCP and oncologist • 61.8% and 48.4% in intervention and control, respectively, found SCP useful • Nearly half (48.4%) of the intervention arm found that the coaching from a lay health educator was useful Qualitative: • Varying experience with coordination of care • SCP was more useful to those with less integrated care and low literacy and well-integrated primary and tertiary care • Survivors found SCP useful to learn about side effects and screening
Greenlee et al. (2016)	Hispanic	*n* = 126 Breast	24-page National Cancer Institute guide called in English and Spanish—“Facing Forward: Life after Cancer Treatment” (intervention and control) Nurse-provided treatment summary and surveillance recommendations based on the ASCO guidelines (intervention) Nutritionist-provided personalized lifestyle recommendations based on nutrition and physical activity guidelines developed by the American Institute for Cancer Research and the American Cancer Society (intervention)	Attitudes towards lifestyle behaviors Knowledge of lifestyle behaviors Frequency of lifestyle behaviors	Quantitative; RCT	• The intervention changed lifestyle behaviors and knowledge in the short-term, but the benefits did not persist • The intervention was more effective among non-Hispanics than among Hispanics in improving attitudes towards healthy eating and frequency of physical activity
Hershman et al. (2013)	Spanish-speaking Hispanic	*n* = 126 Breast	24-page National Cancer Institute guide called in English and Spanish—“Facing Forward: Life after Cancer Treatment” (intervention and control) Nurse-provided treatment summary and surveillance recommendations based on the ASCO guidelines (intervention) Nutritionist-provided personalized lifestyle recommendations based on nutrition and physical activity guidelines developed by the American Institute for Cancer Research and the American Cancer Society (intervention)	Cancer worry Treatment satisfaction Cancer survivor concerns Depression Impact of cancer	Quantitative; RCT	• No significant improvements in patient-reported outcomes • Associated with decreased worry among cancer survivors • Hispanics had higher physical and functional well-being, worse scores on depression scales, and higher scores on worry than non-Hispanics • Hispanics had higher medical trust and higher value on social relationships than non-Hispanics
Kantsiper et al. (2009)	Black/African-American or minority	*n* = 52 (21 survivors, 4 AA survivors, 15 PCPs, 16 oncology specialists) Breast	No specific SCPs evaluated	Needs and priorities of breast cancer survivors, oncology specialists, and PCPs	Qualitative; focus groups	• Survivors viewed an SCP to be shared between providers as valuable to their care coordination • However, it does not replace the active participation of oncology specialists • Oncologists felt that a written SCP or tool to be shared with other providers would be valuable
Kenzik et al. (2016)	Low educational attainment Low income	*n* = 441 (over 65 years) Breast, prostate, lung, colon/rectal, ovarian	No specific SCPs evaluated Assessed receipt of written treatment summaries and follow-up care plans	Self-efficacy ER visit Hospitalization	Quantitative; cross-sectional	• 40% received both written treatment summary and written follow-up plan • 79% of those who received written treatment summaries of follow-up plans received a verbal explanation • Female sex, belonging to minority race/ethnicity, having greater comorbidity were independently associated with self-efficacy (when assessing the relationship between written treatment summary, written follow-up plan, verbal explanation with self-efficacy) • Verbal explanation of follow-up plan associated with decreased healthcare utilization through higher self-efficacy
Ko et al. (2020)	Latina Rural	*n* = 44 (survivors transitioning to primary care (12), family caregivers (8), and healthcare providers (24) Breast	No specific SCPs evaluated	Perspectives on challenges to survivorship care among survivors in the Mexico-US border region	Qualitative; community-based participatory research	• SCP was perceived as useful • SCPs a good source of info to improve survivorship care • SCPs facilitate patient-provider communication • Providers noted SCP could enhance understanding of cancer diagnosis and medication management • SCPs help patients be proactive and aid in formulating questions for the visit • SCPs perceived as reducing psychological distress • SCPs aid in information retention • SCPs provide information to other providers out of state or country • SCPs improve care coordination between providers • SCPs useful for family caregivers
Kim et al. (2022)	Low educational attainment, unemployed	*n* = 17,626 (respondents from BRFSS) Breast gynecologic, prostate, colon, lung, melanoma/other skin, others	No specific SCPs evaluated	Cancer pain Perceived discrimination Physical activity	Quantitative; cross-sectional	• Follow-up care plans positively associated with cancer pain • Treatment summary not associated with cancer pain • Physical activity and discrimination mediated the association between follow-up care plans and cancer pain • Survivors reporting higher discrimination tended to report less pain when receiving follow-up care plans
Ko et al. (2023)	Latina	*n* = 18 Breast	Culturally matched navigators and oncology nurses explaining SCP, reviewing personalized SCP, and coaching and modeling proactive behaviors using a video and psychosocial needs counseling. Booster telephone call a month later	Feasibility (recruitment, retention, completion of baseline assessment, completion of intervention, completion of post-intervention assessment, self-reported use of SCP with healthcare provider, self-reported sharing of SCP with family) Acceptability of Proyecto Mariposa Knowledge of survivorship issues Survivorship concerns Self-efficacy in patient-provider interaction Self-efficacy to manage chronic disease	Mixed; single-arm study; semi-structured interviews	• Almost all agreed SCP enabled them to be proactive • Changes in self-efficacy in managing chronic disease • Information provision—enhanced knowledge about cancer and survivorship care. Video and in-person explanation enhanced understanding • Patient proactiveness—SCP empowered to be proactive, SCP intervention improves family knowledge, intervention encouraged asking questions • Video increased comprehension, interest, and attention to messages
Lewis‐Thames, Marquita et al. (2020)	Rural	*n* = 90 Not specified	No specific SCPs evaluated	Patient-provider communication quality Written communication Timely posttreatment follow-up	Quantitative; cross-sectional	• Survivors receiving written communication had greater odds of reporting timely follow-up care
Maly et al. (2017)	Low income Latina	*n* = 212 Stage 0 to stage III breast cancer	Individually tailored treatment summaries and survivorship care plans (TSSP) TSSP combined with nurse counseling sessions: • Nurse was trained, bilingual, bicultural • Reviewed components of TSSP	Physician implementation of recommended breast cancer care Adherence to recommendations by patients at 12 months	Quantitative; RCT	• SCPs positively impacted physician implementation of recommended breast cancer care • Per subset analysis, Latina patients more likely to agree: ▪ SCP provides more information than its provider ▪ SCP provided information they had not found on their own ▪ Improved communication with their doctors
Nápoles et al. (2019)	Spanish-speaking Latina	*n* = 23 Non-metastatic breast cancer	ASCO SCP template Adapted for low-literacy, Spanish-speaking Latinas Layout simplified and translated to Spanish	Cancer-related fatigue and cancer-related distress Global knowledge of survivorship care Self-efficacy Emotional well-being and somatic symptoms Average daily steps Perceived usefulness of intervention components Perceived ease of use Perceived benefits	Mixed; single-arm study; semi-structured interviews	• Lower fatigue and health distress levels • Greater knowledge of recommended follow-up care and resources • Improvement in emotional well-being • Increased average daily step count • Positive attitudes towards the survivorship care planning program • Survivors reported benefits to knowledge, and well-being in interviews as well • Feeling of a sense of accountability • Shift from extrinsic to intrinsic motivation to be physically active
Shay et al. (2019)	Low educational attainment	*n* = 1855 (respondents from BRFSS) Breast Cervical Colorectal Melanoma Prostate	Paper SCP (written treatment summary and follow-up plan) Association of receipt of written SCPs with health behaviors	Attending a recent medical appointment Exercise in the past month Non-smoking status Mammography in the past 2 years Up-to-date colorectal screening	Quantitative; cross-sectional	• 37% received written SCP • Receipt of SCP independently associated with attending medical appointments, exercise in the past month, non-smoking status, and mammography in the past 2 years • Receipt of SCP not associated with up-to-date colorectal screening
Stewart et al. (2022)	Rural	*n* = 107 (clinicians working in primary care networks; 35% were in rural practices)	Re-engineered sample care plan based on existing EHR-based SCP template	Clinician satisfaction Perceived usefulness	Quantitative; cross-sectional	• Re-engineered SCP was found to be useful, relevant, easy to understand, and in appropriate order • Most participants reported the SCP included the information they wanted to know about their patient’s survivorship care and helped provide better care
Trosman et al. (2021)	Safety net hospitals (5/10 safety net institutions)	*n* = 888 (422 in the intervention cohort, 466 in the historical cohort) Breast	A structured care plan including cancer summary, care checklist, timing, and sequence of interdependent care	Usefulness of 4R Oncology— patient-facing care planning Care Sequence Patient self-management (know the stage of cancer, clarity of care plan, timing and sequence of care clear, seldom or never overwhelmed, not in control of care, able to manage and organize care well or very well, able to explain care to others well or very well, able to express preferences in care well or very well) Care delivery metrics (PCP consult, genetic consult, smoking cessation initiation, dental consult, fertility consult, flu vaccination)	Quantitative; cohort study	• Perceived as useful • Usefulness higher in safety-net than on-safety net patients • 5/7 self-management metrics greater in the intervention group (except able to explain care to others well or very well, able to express preferences in care well or very well) • Referrals to all six types of care were greater in the intervention cohort • Significantly higher referral completion was seen in 4 types of referrals in the intervention cohort • Patients reported components of Care Sequences as useful ▪ Time/sequence graph (69.2%) ▪ Responsibilities (85.2%) • Safety-net hospital patients were more likely to report Care Sequences as useful ▪ Had a greater sense of control and ability to manage their care ▪ Had lesser ability to explain their care when compared with non-safety-net

In place of clinic-based interventions, Casillas and colleagues developed and tested a culturally tailored intervention that included family members of Latino adolescent and young adult survivors and a community health advocate. This intervention resulted in increased knowledge and confidence in the management of survivorship care for both survivors and family members [30]. In another study, Nápoles and colleagues combined a culturally tailored paper SCP, Spanish language mobile health (mHealth) app, and telephone coaching to address survivorship care in Spanish-speaking Latina breast cancer survivors [31]. This intervention reduced fatigue and distress among survivors, increased knowledge of care resources, increased physical activity, improved general well-being, and provided a sense of accountability and motivation through feedback from the telephone coaching and mHealth app [31]. Baseman and colleagues explored the acceptability and feasibility of an SCP in the form of an mHealth app for rural survivors [32]. Both survivors and their providers noted greater self-efficacy and communication on account of the journaling and reports features. Participants in this study also reported that the reminders feature was useful for surveillance [32].

In a survey study of rural cancer survivors, Lewis-Thames and colleagues found that rural survivors receiving written post-treatment survivorship communication had greater odds of reporting timely follow-up care [33]. In an evaluation of a structured care plan called Care Sequence in five safety-net institutions and five non-safety-net institutions, the SCP was perceived as useful [26]. Patient self-management metrics like clarity on the timing and sequence of care, as well as care delivery metrics like flu vaccinations, were higher in those who received Care Sequence. These benefits were similar for both safety-net and non-safety-net survivors. Survivors also cited benefits like having structure to stay focused on the task, and ability to see a timeline, and proactively seeking answers [26]. Similar benefits were mentioned by healthcare providers and survivors in the qualitative study by Ko and colleagues which found the SCPs helped patients become proactive about their care and formulate questions for their healthcare visits [34].

### Theme 2: Receipt of survivorship care plans

Sixteen studies brought attention to cancer survivors and their likelihood of receiving an SCP (see Table 2). A majority of these studies were quantitative using cross-sectional designs (*n* = 13) with two studies using qualitative methods in the form of focus groups (*n* = 1) and semi-structured interviews (*n* = 1). Lastly, one study used both a cross-sectional design as well as semi-structured interviews (*n* = 1). Using a sample of cancer survivors from the LIVESTRONG Survivorship Center of Excellence Network sites, Casillas and colleagues used survey data and found that minority cancer survivors were significantly less likely to have an SCP [35]. In addition, minority cancer survivors had higher odds of reporting low confidence in managing their cancer survivorship care [35].

**Table 2:** Receipt of survivorship care plans among underserved populations (*n* = 16)

Authors	Underserved population	Sample and cancer type	SCP details	Outcomes	Study design	Key findings
Alford-Teaster et al. (2023)	Rural	*n* = 74 (23 survivors, 51 healthcare providers in primary care (*n* = 11) and oncology (*n* = 40)) Breast	Not available	Perspectives on survivorship care planning and use of telehealth	Mixed; cross-sectional; semi-structured interviews; focus group discussions	• 67% of survivors reported not receiving SCP
Arana-Chicas et al. (2023)	Rural	*n* = 27 Solid tumors	Not available	Needs of older rural cancer survivors	Qualitative; semi-structured interviews	• Most survivors did not receive an SCP • Survivors reported losing appetite and energy • Transportation challenges reported • Psychological challenges of undergoing chemotherapy • Financial toxicity
Casillas et al. (2011)	Minority Low educational attainment	*n* = 376 Leukemia/lymphoma, bone and soft tissue sarcomas	No specific SCPs evaluated	Self-reported receipt of survivorship care planning Expectations from providers Confidence in managing survivorship care	Quantitative; cross-sectional	• Ethnic minority survivors were associated with higher odds of lacking a survivorship care plan
Desmond et al. (2017)	Low educational attainment Low income	*n* = 1105 (BRFSS respondents) Non-melanoma skin cancer	No specific SCPs evaluated	Survivorship characteristics Received written summary of cancer treatments	Quantitative; cross-sectional	• No significant differences found in the receipt of treatment summary by race • Survivors with higher education and higher income were more likely to report receiving instructions from a doctor for follow-up care
Hinyard and Wirth (2017)	Non-Hispanic Black and Asian	*n* = 1185 (sampling strategy is such that non-Hispanic black and Asian adults over 65 have greater probability of selection) Colon, breast, melanoma/other skin, gynecologic, others	No specific SCPs evaluated	Provision of written advice pertaining to routine follow-up care Provision of written information on cancer treatments Odds of receiving both written plans or one written plan versus receiving none	Quantitative; cross-sectional	• Race was the strongest predictor of receiving either type of written plan
Jabson and Bowen (2013)	Low educational attainment Low income	*n* = 6897 Colon, breast, melanoma/other skin, gynecologic, others	No specific SCPs evaluated	Written treatment instructions Follow-up care instructions Written follow-up care instructions	Quantitative; cross-sectional	• Lower income and education associated with not receiving written treatment summary and follow-up care instructions • Hispanic survivors more likely to receive treatment summaries
Ko et al. (2021)	Black	*n* = 53 Breast	No specific SCPs evaluated	Needs and experiences	Qualitative; focus groups	• Participants reported supportive relationships with their PCPs • They discussed building relationships with their oncology providers • Survivors did not receive consistent survivorship planning, many receiving only verbal instructions • Half did not receive written or electronic SCP or do not remember • They also discussed positive and negative experiences associated with getting diagnosed and receiving care
Linscott et al. (2020)	Low educational attainment Low income	*n* = 2416 (BRFSS respondents) Bladder, kidney, prostate	No specific SCPs evaluated	Survivorship care plan receipt	Quantitative; cross-sectional	• Low-income patients and lower-education patients were less likely to receive a survivorship care plan
Malhotra et al. (2022)	Non-Hispanic Black	*n* = 112 Lung	No specific SCPs evaluated	Receipt of recommended surveillance scans and follow-up care (including receiving treatment summary)	Quantitative; cross-sectional	• 57% of survivors received treatment summary • No difference in receipt of treatment summary by race
Millar et al. (2023)	Uninsured, Hispanic	*n* = 1793 (oversampled if living in areas with high proportions of uninsured residents and Hispanic ethnicity) Breast, prostate, melanoma, colorectal, thyroid	No specific SCPs evaluated	Health indicators like smoking, physical activity, pain, health status, clinical are covered by insurance, experience limitations due to physical emotional, or mental problems, participated in clinical trial, dissatisfied with life, receipt of written SCP	Quantitative; cross-sectional	• 40.4% received written SCP with both components, no differences by ethnicity • 68.2% received follow-up instructions alone • 51.2% received treatment summary alone
Rencsok et al. (2022)	Non-Hispanic Black	20% Black Prostate cancer	No specific SCPs evaluated	Receipt of written assessment plan Receipt of the name of non-physician personnel for support Being involved as much as they want in care Feeling like their views were taken into account	Quantitative; cohort	• 71% Black survivors received a written care plan, and 58% White survivors received written care plan • Higher prevalence among Black survivors to receive information about treatment • Integration with care and respect for preferences
Sabatino et al. (2013)	Low educational attainment Low income	*n* = 1345(NHIS respondents) Breast, prostate, cervix, melanoma, colorectal, uterus	No specific SCPs evaluated	Receipt of treatment summary Receipt of follow-up instructions	Quantitative; cross-sectional	• 22% received both treatment summaries and written follow-up instructions • 45% did not receive either • Black survivors were more likely than White survivors to receive treatment summary as well as written follow-up instructions • Lower income associated with a lower likelihood of receiving written follow-up instructions
Shay et al. (2019)	Low educational attainment	*n* = 1855 (respondents from BRFSS) Breast Cervical Colorectal Melanoma Prostate	Paper SCP (written treatment summary and follow-up plan) Association of receipt of written SCPs with health behaviors	Attending a recent medical appointment Exercise in the past month Non-smoking status Mammography in the past two years Up-to-date colorectal screening	Quantitative; cross-sectional	• 37% received written SCP • Receiving written SCP associated with higher education, being uninsured, type of cancer, provider type, exercise in the past month, up-to-date mammography
Tawfik et al (2021)	Rural Low-income minority	*n* = 283 (comprehensive cancer center treating poor, rural, and minority cancer patients) Breast, gynecological, prostate, colorectal, lymphoma	SCP templates based on ASCO and SGO templates SCPs were delivered electronically and integrated with the EHR SCPs were also faxed or mailed	Time points: time from cancer diagnosis to SCP ordered, SCP ordered to SCP created, SCP created to hardcopy SCP delivered to patient by provider, and rate and route of delivery of SCPs to PCPs Receipt and integration of SCPs by PCPs	Quantitative; cross-sectional	• 85.2% of SCPs created of those ordered • 34.2% of created SCPs given to patients
Timsina et al. (2021)	Low educational attainment	*n* = 7061 (BRFSS respondents) Not specified	No specific SCPs evaluated	Receipt of SCP (receiving both treatment summary and follow-up care instructions after completing treatment)	Quantitative; cross-sectional	• Lower educational attainment and being uninsured decreased the probability of the receipt of SCP • There was no association between race/ethnicity and receipt of SCP
Wu et al. (2018)	Low income Low educational attainment	*n* = 1446 (BRFSS respondents) (492 with gynecologic cancers, 954 with breast cancer) Breast, ovarian, endometrial, and cervical cancers	No specific SCPs evaluated	Receipt of instructions on follow-up care	Quantitative; cross-sectional	• Odds of receiving follow-up instructions associated with type of cancer, income, and BMI • Race did not modify the association between cancer type and receipt of follow-up instructions

Several cross-sectional studies using Behavioral Risk Factor Surveillance System (BRFSS) data also showed that cancer survivors with lower education [36–39] and income [36–39] and being uninsured [39] were less likely to report receiving SCPs. For example, Jabson and colleagues found that demographic characteristics were associated with the receipt of treatment summaries and follow-up care instructions [37]. More specifically, cancer survivors who reported completing less than a high school education and who reported household incomes lower than $50,000 had lower odds of reporting receipt of treatment summaries. Incidentally, this study also found that Hispanic cancer survivors were more likely to receive treatment summaries [37]. Wu and colleagues used BRFSS data and found that low-income breast cancer patients had lower odds of receiving follow-up care instructions from their providers [40].

Sabatino and colleagues examined the receipt of treatment summaries and follow-up instructions among African-American and Hispanic cancer survivors [41]. This study found that many recently diagnosed cancer survivors did not report receiving treatment summaries and written follow-up instructions. In addition, race and ethnicity were associated with lower reporting of summaries. DeGuzman and colleagues explored the needs of a sample of seven rural, low-income breast cancer survivors and their post-treatment survivorship care planning [18]. This study found that rural survivors had a lack of knowledge of post-treatment care, such as how to assess cancer recurrence. In addition, none of the women reported receiving or hearing about an SCP. While some women reported receiving a packet of information, there was no specific information provided.

Tawfik and colleagues implemented and evaluated a process of generating and delivering SCPs to patients and providers in a comprehensive cancer center that serves poor, rural, and minority cancer patients [42]. They reported that of all the SCPs ordered, about 85% were generated, and of those generated about a third (34.2%) were given to patients. Approximately half (48.9%) of SCPs were sent to primary care providers (PCP) by mail or fax, and 8.3% of these were received [42].

### Theme 3: Needs and preferences of survivors

A third major theme was related to identifying the unmet needs and preferences of groups that are underserved with respect to the contents of SCPs. We identified 18 studies targeting Black, Latina, rural, and low-income cancer survivors (see Table 3). These studies were predominantly qualitative (*n* = 12) followed by 5 mixed-methods studies and 1 quantitative study. Most studies used focus groups (*n* = 5) or semi-structured interviews (*n* = 3). Others included CBPR (consensus meetings, focus groups, interviews, RAND-Delphi methods) (*n* = 3) and a think aloud study (*n* = 1). The mixed method studies were cross-sectional (*n* = 3) or single-arm trials (*n* = 2), and most used semi-structured interviews as well (*n* = 4).

**Table 3:** Needs and preferences of survivorship care plans (*n* = 18)

Authors	Underserved population	Sample and cancer type	SCP details	Outcomes	Study design	Key findings
Alford-Teaster et al. (2023)	Rural	*n* = 74 (23 survivors, 51 healthcare providers in primary care (*n* = 11) and oncology (*n* = 40)) Breast	Not available	Perspectives on survivorship care planning and use of telehealth	Mixed; cross-sectional; semi-structured interviews; focus group discussions	Content: Areas of information deficits: • Side effects • Access to mental health services needed Format: • Survivors also find that there is a lot of information and paper • Survivors reported access and comfort of use with telehealth (clinician and survivor reports conflicted regarding telehealth access)
Arana-Chicas et al. (2023)	Rural	*n* = 27 Solid tumors	Not available	Needs of older rural cancer survivors	Qualitative; semi-structured interviews	Content: Areas of information deficits: • Side effects • Guidance on diet and exercise • Referrals to support groups SCP preferences: • One-on-one discussion of survivorship care
Ashing-Giwa et al. (2013)	Black/African American	*n* = 28 (25 survivors, 3 advocates) Breast	ASCO SCP template • Cancer-related information (i.e., treatment history, medication, side effects) • Follow-up care/surveillance recommendations • Health advisories • Quality of life information	Understanding of SCP Feedback on cultural and socio-ecological responsiveness	Qualitative; community-based participatory research (consensus meetings)	Participants felt they would benefit from a well-organized document such as an SCP Content: Areas of information deficits • Health history • Comorbidity • Health promotion • Functioning • Genetic testing • Recurrences • Symptom management • Management of lifestyle factors like nutrition and physical activity (relevant to AAs) • Spiritual care referrals and resources • Informational, medical, and supportive care resources • Sexual side effects • Referrals to culturally competent providers Format: • Cover page highlighting the relevance of SCP • Usability can be improved by having more space between items • Readability can be improved by enlarging the text • Space should be provided for documenting recurrences • Space to note which treatment regimens worked • Section to note referrals with specialists
Ashing et al. (2014)	Latina	*n* = 22 (12 survivors;10 stakeholders) Breast	ASCO Breast Cancer Adjuvant Treatment Plan and Summary and Survivorship Care Plan (TSSCP) template • Cancer-related information (i.e., treatment history, side effects) • Follow-up care/surveillance • Health advisories • HRQOL information	Inform development and evaluation of tailored SCP The Treatment Summary and Survivorship Care Plan (TSSCP-S)	Mixed; cross-sectional; community-based participatory research (consensus meetings)	TSSCP-S was found to be patient-user-friendly and easy to understand The tailored SCP achieved clinical, cultural, linguistic, and social responsiveness SCP preferences: Survivors received the following favorably: • Bilingual (Spanish and English) • Amount and quality of detail Content: Areas of information deficits: • Cultural values (familism, trust, respect) • Social practices (spirituality, family support) • Nutrition and physical activity guidelines • Supportive care resources • Health advisories • Health-related quality of life (HRQoL) • Survivorship resources that do not need Internet access • Introduction and narrative for PCPs and survivors Format: • Spanish language translation adjacent to English text • Images • Preference for print SCP
Baseman et al. (2017)	Rural	*n* = 11 (6 survivors, 4 primary care providers, 1 oncologist) Breast	Mobile phone app (SmartSurvivor) and website: • Care team • Treatment summary (diagnosis, radiation treatment summaries, etc.) • Follow-up care (e.g., ongoing toxicities to track, wellness/ concerns, recommended follow-up schedule/frequency, etc.) • Tracking tool for self-monitoring	Feasibility and Acceptability of SCP as mHealth app (SmartSurvivor)	Qualitative; think aloud	Further tailoring desired for rural survivors regarding readability Format: • Survivors did not want paper SCPs Content: • Providers found the reminders function useful if the reminders could be recalibrated easily in case of missed appointments particularly for rural survivors • Providers thought background information would be helpful in deciding if routine appointments could be done locally than with specialists who were far away SCP aids: Providers recommended: • Photos • Visual cues • Simplified language for patient education material on the SCP
Burg et al. (2009)	African American	*n* = 32 Breast	ASCO SCP template • Breast cancer adjuvant treatment summary • Breast cancer survivorship care plan • Recommendations for follow-up care (e.g., medical history and physical examination, post-treatment mammography, breast self-examination, pelvic examination, coordination of care and genetic counseling referral) • Follow-up frequency list of symptoms of recurrence	Types of survivorship information received Opinions on value and content of SCPs	Qualitative; focus groups	Participants believed all survivors should receive SCPs Content: Areas of information deficits: • Care • Follow-up • Side-effects • Nutrition • Exercise • Resources for information and support Concerns with SCPs included: • Treatment summary contains a lot of medical jargon • Generic information on surveillance • Information should be discussed with a healthcare professional
Burke et al. (2016)	Safety-net Minority	*n* = 38 Breast	No specific SCP discussed	Informational and structural challenges to treatment and survivorship care for safety-net breast cancer patients Preferences for content and delivery of SCPs	Qualitative; focus groups	Timing and delivery: Survivors wanted SCP delivered at transition points: • During treatment • Between active treatment and survivorship • Subsequent points when needed Content: Areas of information deficits: • Incorporating information specific to hereditary breast cancer as part of SCPs • Information on how to talk to family about living with cancer • Referrals to PCPs, who are knowledgeable about breast cancer and side effects • Screening • Recurrence • Side effects and pain • Lymphedema • Reconstruction • Healthy eating/physical activity
Casillas et al. (2022)	Latino	*n* = 34 (adolescents and young adults, 16 parents and family members) Leukemia, lymphoma, brain/central nervous system, bone, soft tissue	Photonovela survivorship story Actionable survivorship care planning tools Sample customizable SCP	Develop and test the acceptability of a culturally appropriate photonovela intervention to improve knowledge of, and the need to receive, survivorship care	Qualitative; community-partnered participatory research (RAND-Delphi method, focus groups)	All participants received the photonovela positively Timing and delivery: • Best time for receiving SCP information was remission not diagnosis Format: • Highlighting components of an SCP SCP aids: • All liked the peer explanation of an SCP
DeGuzman et al. (2017)	Rural, low-income	*n* = 28 Breast	No specific SCP discussed	Post-treatment survivorship care planning execution Perception and needs of rural, low-income cancer survivors	Qualitative; semi-structured interviews	None of the participants was aware of SCPs Rural survivors’ responses reflected a lack of knowledge about post-treatment care, including how to assess for cancer recurrence Format: • Survivors reported receiving packets but not using them Content: Areas of information deficits • Non-medical supportive care needs • Changes related to reconstructive surgery Timing and delivery: • Timing of SCP discussion is critical. Final treatment appointment may not be ideal for retention (for rural patients)
Ko et al. (2020)	Latina	*n* = 44 (40 focus group participants, 4 interview participants; breast cancer survivors, family caregivers, nurses, medical assistants, social work patient navigators, physicians) Breast	No specific SCPs evaluated	SCP and SCP aid needs and preferences	Qualitative; community-based participatory research	Format: • Paper SCP SCP aid preferences: • Animated video • Patient navigators to provide educational information in SCP
Ko et al. (2021)	Black	*n* = 53 Breast	No specific SCPs evaluated	Needs and experiences	Qualitative; focus groups	Content: Areas of information deficits: • Some side effects interfered with quality of life more than others • Recurrence • Body image challenges • Financial toxicity and the financial strain of treatment • Spirituality helped in coping • Symptoms • Medication side effects like weight gain and fertility • Diet and physical activity that is culturally tailored Format: • Preference for video material over reading material
Ko et al. (2023)	Latina	*n* = 18 Breast	Culturally matched navigators and oncology nurses explaining SCP, reviewing personalized SCP, coaching and modeling proactive behaviors using a video, psychosocial needs counseling Booster telephone call a month later	Feasibility (recruitment, retention, completion of baseline assessment, completion of intervention, completion of post-intervention assessment, self-reported use of SCP with healthcare provider, self-reported sharing of SCP with family) and Acceptability of Proyecto Mariposa Knowledge of survivorship issues Survivorship concerns Self-efficacy in patient-provider interaction Self-efficacy to manage chronic disease	Mixed; single-arm study; semi-structured interviews	Survivors appreciated the information in the SCP SCP aids: • In-person sessions facilitated better understanding of the SCP • Video increased comprehension, interest, and attention to message
Nápoles et al. (2019)	Spanish-speaking Latina	*n* = 23 Non-metastatic breast cancer	Spanish-language mobile app (trackC) • Women’s breast cancer diagnostic and treatment history • Information on potential side effects • Healthy lifestyles • Survivorship resources	Cancer-related fatigue and cancer-related distress Global knowledge of survivorship care Self-efficacy Emotional well-being and somatic symptoms Average daily steps Perceived usefulness of intervention components Perceived ease of use Perceived benefits	Mixed; single-arm study; semi-structured interviews	Content: Survivors appreciated: • Information on disease • Ability to track Format: • Survivors preferred SCP in both written and app format SCP aids: • Motivation from check-ins with a coach • Appreciated tailoring by coach • Appreciated visual and auditory feedback on the app (like applause on achieving a health goal)
Rutledge et al. (2017)	Rural Minority	*n* = 53 (survivors and PCPs) Endometrial	No specific SCPs evaluated	Insights on transitioning to primary care from oncology Content and format of SCPs	Qualitative; focus groups	Survivors and providers supported the use of individualized SCPs Format: • Providers preferred SCPs in paper and electronic format • Ability to share with all care team members Content: Areas of information deficits: • Information on exercise and nutrition • More information on transitioning to primary care
Tevaarwerk et al. (2022)	Rural	*n* = 13 (clinicians) Not specified	EHR-based SCP template Assessed preferences for SCP elements	SCP information needs and preferences Usefulness of 16 SCP elements	Mixed; cross-sectional; semi-structured interviews	Content: • Recommended cancer-related screening • Preventative screening impacted by cancer/treatment • Timeline for recurrence risk • Prioritizing follow-up care over treatment summaries • Removing or down-playing screening recommendations not cancer-related • Annual updates • Information on lifestyle and psychosocial resources was considered less useful • Vaccination recommendations • Genetic testing • Stating “not recommended” for unnecessary screening to validate PCPs’ decisions Format: • Single SCP for patients and clinicians • Electronic format Layout/design • Labeling of SCP sections with survivor name and “survivorship care plan” • Addition of date created field below headers to facilitate determining outdatedness • Clearly labeling cancer diagnosis as early as possible • Moving abbreviated clinical contact information to the front along with provider-to-provider communication • Direct links from treatment information to supporting documents
Tisnado et al. (2016)	Latina	*n* = 74 Breast	No specific SCPs evaluated	Knowledge, attitudes, and beliefs regarding survivorship care Experiences with survivorship care activities	Qualitative; focus groups	• All participants expressed a desire for a formalized SCP • The few women who received an SCP were treated at high-resource cancer centers • Most of these women received follow-up care plans rather than treatment summaries
Trosman et al. (2021)	Safety net hospitals (5/10 safety net institutions)	*n* = 888 (422 in the intervention cohort, 466 in the historical cohort) Breast	A structured care plan including cancer summary, care checklist, timing, and sequence of interdependent care	Usefulness of Care Sequence Patient self-management (know stage of cancer, care plan clear or very clear, timing and sequence of care clear, seldom or never overwhelmed, not in control of care, able to manage and organize care well or very well, able to explain care to others well or very well, able to express preferences in care well or very well) Care delivery metrics (PCP consult, genetic consult, smoking cessation initiation, dental consult, fertility consult, flu vaccination)	Quantitative; cohort study	SCP aids: Patients reported components of Care Sequences as useful • Time/sequence graph (69.2%) • Responsibilities (85.2%) Format: • 47.2% preferred paper format • 40.3% preferred paper format with an electronic copy (6.6% preferred electronic copy) • Having key information on one sheet
Wen et al. (2014)	Minority	*n* = 16 Breast	No specific SCPs evaluated	Chinese American breast cancer survivors’ perceptions of SCPs Preferences for content and format of SCPs	Qualitative; semi-structured interviews	All participants noted that an individualized SCP would be useful Content: Survivors appreciate these elements as useful: • Diagnosis • Treatment summary • Information on recurrence • Side effects • Lifestyle Areas of information deficits: • Traditional Chinese medicine • Culturally relevant lifestyle recommendations Format: • Information in lay language, both in Chinese and English • Preference for written SCP SCP aids: • In-person review of SCP with provider • Cultural differences influence provider-patient communication • Survivors in this study reported using family members as translators (role of professional interpreters)

#### Content

In a qualitative study seeking to understand if SCPs are responsive to the needs of Black breast cancer survivors, Ashing-Giwa and colleagues found that while Black cancer survivors felt they would benefit from a well-organized SCP, they found several limitations to the SCP including inadequate information related to health history, the number and severity of comorbidities, health promotion, referrals to other specialists, and functioning [43]. Similar findings were presented in other qualitative studies [44, 45]. Burke and colleagues found that racial and ethnic minority women suggested that the SCP should include referrals to PCPs who were knowledgeable about their cancer and associated side effects. Women in this study also hoped the SCP would provide them with questions to ask their PCP [45].

The preferences for content differed based on the survivor or provider perspective. For example, minority cancer survivors noted wanting more information on lifestyle management, like physical activity and nutrition [43–46], whereas providers noted this type of content was less useful [47]. However, both groups agreed that information on genetic testing was an important addition [43, 45, 47]. Other needs expressed by Black survivors included referrals to spiritual care and culturally competent providers [43].

Tisnado and colleagues found numerous gaps and unmet needs among a sample of Latina breast cancer survivors [48]. While few women in this sample reported receiving an SCP, those who did receive an SCP were treated at a high-resource cancer center. In addition, participants also reported unmet needs in their survivorship care related to finances, continuity of care, and a lack of information related to symptom management.

Studies also assessed perspectives on the different components of SCPs. Preferred SCP elements included graphical representation of timing and sequence of care [26] and visual and auditory feedback from SCPs embedded in apps [31].

#### Format

In terms of format, survivors differed in their preference for paper versus electronic SCPs. Hispanic breast cancer survivors preferred a print SCP [34, 46]. Conversely, rural participants in the study by Baseman and colleagues, which examined the feasibility and acceptability of SCP as an mHealth app, reported they did not want print SCPs [32]. In addition, rural PCPs preferred an electronic SCP that could be pushed from the oncologists’ electronic health record (EHR) to theirs [47].

Other format suggestions included the use of images [32, 46], improved readability [32, 44, 46], and tailoring for cultural appropriateness [43, 46]. Several preferences for layout were articulated across these studies: having key information on one sheet [26] and providing space for Black survivors to make notes [43].

#### Facilitating conditions

In addition to content, format, and layout, survivors mentioned other factors that would facilitate the use of SCPs.

##### SCP aids

For example, Hispanic survivors identified SCP aids in the form of patient navigators or coaches [31, 49]. Black survivors believed the information shared within SCPs should be discussed with their healthcare providers [44]. Studies also found that Hispanic patients also preferred aids like animated videos [34] or photonovelas [30] to provide SCP information.

Findings based on survivor perspectives suggest that discussing SCPs with the provider, and the timing when this discussion occurs, may be associated with the implementation of SCPs. Burke and colleagues reported that in-person review of SCPs, and delivery of SCPs at transition points in the cancer journey such as during active treatment, between active treatment and survivorship may facilitate the use of SCPs [45]. However, DeGuzman and colleagues reported that discussing SCPs in the final treatment appointment hindered knowledge retention by survivors [18].

##### Language

In a project aiming to translate and tailor the ASCO breast cancer treatment summary template to Latino breast cancer patients, the translated version was found to be more favorable than the ASCO template with respect to content, clarity, utility, cultural and linguistic responsiveness, and socioecological responsiveness [46]. Participants also liked that the template was bilingual, providing English text with Spanish translations adjacent to each other.

##### Coordination

Both survivors and providers noted that the transition from cancer care to primary care was poor [47]. Survivors suggested that SCPs include referrals to PCPs knowledgeable about cancer side effects [45].

### Theme 4: Barriers and facilitators to implementation of survivorship care plans

Lastly, we identified 19 studies related to barriers and facilitators of implementing SCPs predominantly based on healthcare provider perceptions (see Table 4). Seven of these studies were qualitative, 4 were quantitative, and 8 used mixed methods. The qualitative studies used semi-structured interviews (*n* = 3), focus groups (*n* = 2), CBPR (consensus meetings) (*n* = 1), and think aloud approaches (*n* = 1). Half of the quantitative studies were cross-sectional (*n* = 2), and the rest were a cohort study (*n* = 1) and an RCT (*n* = 1). Of the 8 studies using mixed methods, 3 were cross-sectional, 1 was an RCT, 3 were single-arm studies, and 1 carried out a suitability of materials assessment. The qualitative components included semi-structured interviews (*n* = 5), focus groups (*n* = 1,) and CBPR (consensus meetings) (*n* = 1). This theme is further divided into 4 categories.

**Table 4:** Barriers and facilitators of survivorship care plans in the healthcare setting (*n* = 19)

Authors	Sample	Cancer type	SCP details	Outcomes	Study design	Barriers	Facilitators
Alford-Teaster et al. (2023)	Rural	*n* = 74 (23 survivors, 51 healthcare providers in primary care (*n* = 11) and oncology (*n* = 40)) Breast	Not available	Perspectives on survivorship care planning and use of telehealth	Mixed; cross-sectional; semi-structured interviews; focus group discussions	• SCP is liked but PCPs do not receive them • Survivors do not transition back to primary care as they do not share a relationship with their PCP • Clinicians thought shared telehealth appointment was unrealistic in their current care model	• Survivors and clinicians supported shared telehealth appointments for survivorship care planning
Arana-Chicas et al. (2023)	Rural	*n* = 27 Solid tumors	Not available	Needs of older rural cancer survivors	Qualitative; semi-structured interviews	• Feeling overwhelmed with the paperwork and terminology • Lack of coordination between PCP and oncology • Poor understanding of work-related challenges • Lack of consideration for farm animals that survivors work with	• One on one discussion of survivorship care
Ashing-Giwa et al. (2013)	Black/African American	*n* = 28 (25 survivors, 3 advocates) Breast	ASCO SCP template • Cancer-related information (i.e., treatment history, medication, side effects) • Follow-up care/surveillance recommendations • Health advisories • Quality of life information	Understanding of SCP Feedback on cultural and socio-ecological responsiveness	Qualitative; community-based participatory research (consensus meetings)	• Scheduling additional appointments to complete SCP or pay for staff time to complete SCP	• View the entire SCP with doctors • Survivors and providers complete relevant sections of the SCP • Survivor and PCP complete sections on family history, comorbidity, HRQOL, and advisories • Oncology team completes section on cancer treatment, follow-up treatment, medical referrals, HRQOL, and advisories
Ashing et al. (2014)	Latina	*n* = 22 (12 survivors;10 stakeholders) Breast	ASCO Breast Cancer Adjuvant Treatment Plan and Summary and Survivorship Care Plan (TSSCP) template • Cancer-related information (i.e., treatment history, side effects) • Follow-up care/surveillance • Health advisories • HRQOL information	Inform development and evaluation of tailored SCP	Mixed; cross-sectional; community-based participatory research (consensus meetings)	• Physicians may not have time to complete SCP	• Patient activation achieved through peer support, navigation, and advocacy training • Culturally and linguistically appropriate SCP
Baseman et al. (2017)	Rural	*n* = 11 (6 survivors, 4 primary care providers, 1 oncologist) Breast	Mobile phone app (SmartSurvivor)and website • Care team • Treatment summary (diagnosis, radiation treatment summaries, etc.) • Follow-up care (e.g., ongoing toxicities to track, wellness/concerns, recommended follow-up schedule/frequency, etc.) • Tracking tool for self-monitoring	Feasibility and acceptability of SCP as mHealth app (SmartSurvivor)	Qualitative; think aloud	• Having a paper-based SCP • Concerns about interoperability • Health literacy among rural survivors • Primary care providers noted survivors rarely bring their entire paper SCP upon transition. They carried a subset of the pages occasionally • Patients in rural areas experience unique challenges in survivorship care planning • Lower health literacy among rural patients also presents a barrier to SCP • Data in apps may exist in silos and may not communicate with EHR or clinical data • Data quality of information on the app based on whether entry by hand or through EHR	• One location for all information • Journaling and associated reports feature for symptom tracking • Graphical representation of symptom tracking for seeing trends and patterns and communication with providers • Providers find graphs can be used as an aid in clinical decision-making and save time • Portability • mHealth may help patients in rural areas address challenges with SCP; however, unique needs may need to be addressed • Interoperability of mHealth app with other apps that track diet and exercise may address challenges related to data silos • Timing of reminders/notifications reminders valued when under 3 years post-treatment • Recalibration feature for missed appointments to accommodate rural survivors’ transportation issues • Using photos, visual cues, and simplified language to address the needs of rural survivors • Simplified data entry to record symptoms for tracking, auto-capture of date and time
Burke et al. (2016)	Safety-net Minority	*n* = 38 Breast	No specific SCP discussed	Informational and structural challenges to treatment and survivorship care for safety-net breast cancer patients Inform content and delivery of SCPs	Qualitative; focus groups	• Lack of provider buy-in • Lack of reimbursement • Women wanted SCP delivered at the transition point (e.g., during treatment, between active treatment and survivorship, subsequent points when needed)	• Review of the SCP in person with a provider • SCP should have information in lay language in English • Incorporating perspectives of ethnically and linguistically diverse patients may help with SCP
DeGuzman et al. (2017)	Rural Low income	*n* = 28 Breast	No specific SCP discussed	Post-treatment survivorship care planning execution Perceptions and needs of rural, low-income cancer survivors	Qualitative; semi-structured interviews	• None of the participants were aware of SCPs • Delivery of the SCP during the final treatment appointment was inadequate for knowledge retention • Rural survivors’ responses reflected a lack of knowledge about post-treatment care, including how to assess for cancer recurrence • Lack of time to discuss SCPs, especially with rural patients • Cost may be a barrier for rural patients to commute to care sites • Mode of delivery (pile of papers) • Lack of information on nonmedical supportive care needs • Lack of information on changes related to reconstructive surgery	• Avoid discussing SCP plans in the final treatment to improve retention • Telemedicine may potentially help rural patients connect with providers and should be considered • Additional provider types such as oncology nurses may also address unmet needs among underserved populations
Duggan et al. (2023)	Rural	*n* = 72 Breast, prostate, colorectal, lymphoma	SCP with coaching from a lay health educator (intervention) SCP alone (control)	Health literacy; self-efficacy; quality of life	Mixed; RCT; semi-structured interviews		• Nearly half (48.4%) of the intervention arm found the coaching from a lay health educator was useful • SCP was more useful to those with low literacy and less integrated care
Isaacson et al. (2018)	Rural	*n* = 5 (1 administrator, 4 nursing leaders) Not specified	Health system A Individualized SCP prepared through Microsoft Word-friendly EHR Health system B EHR-integrated SCP templates that can be completed for individual patients on an ongoing basis	SCP development and implementation at two large rural health systems	Qualitative; interviews	• One health system recognized inefficiencies with SCP templates and developed in-house system • Other health systems EHR vendor did not support SCPs • Operability with the EHR system was identified as a challenging aspect • Automatic population of fields on SCPs • Time and cost of implementing SCPs • Type of cancer influences if the patient amenable for SCPs • Difficulty assessing SCP status for patients seeing providers who are not affiliated with health system but perform surgery at health system facility	• Standardization of SCPs sent to PCPs as they receive SCPs from different organizations
Klemp et al. (2022)	Rural	*n* = 57 providers for phase 1 *n* = 90 for phase 2 Not specified	No specific SCP evaluated	PCP perspectives on survivorship care	Mixed; focus groups; single-arm study	• PCP teams reported rarely or never receiving SCP from oncology providers • PCPs did not have a formal process to identify cancer survivors within their practice • Were unaware of how to access resources for cancer survivors • Lack of EHR integration	• 1 to 2 pages with specific recommendations from oncologists
Kantsiper et al. (2009)	African American	*n* = 52 (21 survivors, 4 AA survivors, 15 PCPs, 16 oncology specialists) Breast	No specific SCPs evaluated	Needs and priorities of breast cancer survivors, oncology specialists, and PCPs	Qualitative; focus groups	• PCPs voiced concerns for SCPs that individual plans would need to be periodically updated by oncologists as guidelines changed	• Written SCP shared with other providers • Periodic updates to SCPs by oncologists as guidelines changed
Ko et al. (2023)	Latina	*n* = 18 Breast	Culturally matched navigators and oncology nurses explaining SCP Reviewing personalized SCP Coaching and modeling proactive behaviors using a video Psychosocial needs counseling Booster telephone call a month later	Feasibility (recruitment, retention, completion of baseline assessment, completion of intervention, completion of post-intervention assessment, self-reported use of SCP with healthcare provider, self-reported sharing of SCP with family) and acceptability of Proyecto Mariposa Knowledge of survivorship issues Survivorship concerns Self-efficacy in patient-provider interaction Self-efficacy to manage chronic disease	Mixed; single-arm study; semi-structured interviews		• Survivors appreciated the information in the SCP but also recognized in-person sessions facilitated better understanding of the SCP • Non-physician staff implementing the program • Language-concordant, culturally tailored SCP • Individually tailored SCP • Video format
Lyson et al. (2021)	Vulnerable groups	*n* = 16 (SCPs from different healthcare settings) Breast and colorectal cancer	Multiple different SCPs SCPs from health systems treating vulnerable groups 16 SCPs examined	Extent to which SCPs captured the elements recommended by the IOM Assess overall communication appropriateness for adult patients (readability and cultural appropriateness)	Mixed; suitability assessment of materials	• None of the SCPs were completely IOM concordant • Number of IOM recommended components in the SCP varied by type of healthcare setting • None of the SCPs included whether nutritional, psychosocial, and other supportive services were provided but mentioned the potential need for them • Mean reading grade was 14 • Key information like information on recurrence, future screening, and cancer care key contact missing in some SCPs • No tailoring for any populations besides non-Hispanic Whites • Communication appropriateness also varied by setting (reading level and cultural appropriateness)	• Resources and staff time availability to develop SCPs • Co-designing SCPs with diverse patient populations • Develop Tailored ASCO or Journey Forward SCPs from national organizations to address limited time and resource limitations
Nápoles et al. (2019)	Spanish-speaking Latina	*n* = 23 Non-metastatic breast cancer	Bilingual SCP, Spanish-language app trackC with integrated activity trackers ASCO SCP template for Spanish speakers	Cancer-related fatigue and cancer-related distress Global knowledge of survivorship care Self-efficacy Emotional well-being and somatic symptoms Average daily steps Perceived usefulness of intervention components Perceived ease of use Perceived benefits	Mixed; single-arm study; semi-structured interviews	• Technical issues with app	• Experience with mobile phones • Customization of app information based on hormone status • Low literacy, experience with mobile phones, and vision impairments were challenges • Assistance for survivors with limited mobile phone experience • mHealth can be adapted for auditory and visual impairments and low literacy • Supplemental coaching or individualized assistance on using mobile phones • For medically underserved populations mHealth alone is inadequate. Personal and intensive delivery of intervention needed
Psihogios et al. (2021)	Minority	*n* = 110 Leukemia/lymphoma, solid tumor, brain tumor	Electronic SCP within an app. The app also provides two-way, tailored mobile messages to reinforce and enhance uptake of SCP	Engagement: percentage of active app days, percentage of mobile messages read, SCP views Acceptability: ease of use, perceived usefulness, engagement barriers, facilitators	Quantitative; RCT	• Primary engagement barriers identified were technical glitches (e.g., app crashing, malfunctioning notifications) and competing priorities (e.g., work, college)	• Embedding SCP in an app • Uninterrupted app access • iPhone use • Receiving messages in summer months compared to non-summer months • Certain types of messages such as health goal messages and trivia questions facilitated engagement with the app • Lower depression, higher motivation, and better health perception had a higher percentage of active app days
Stewart et al. (2022)	Rural	*n* = 107 (clinicians working in primary care networks; 35% were in rural practices)	Re-engineered EHR-based SCP template Assess primary care clinician-perceived usefulness of the re-engineered template	Clinician satisfaction Perceived usefulness	Quantitative; cross-sectional	• Lack of EHR integration and access location • SCP length precludes quickly referencing in a clinical setting	• Bulleted list or summary section at the beginning of the SCP • SCP found within the problem list in EHR or another specific area
Tawfik et al. (2021)	Rural Low-income Minority	*n* = 283 Breast, gynecological, prostate, colorectal, lymphoma	SCP templates based on ASCO and SGO templates SCPs were delivered electronically and integrated with the EHR SCPs were also faxed or mailed	Time points: time from cancer diagnosis to SCP ordered, SCP ordered to SCP created, SCP created to hardcopy SCP delivered to patient by provider, and rate and route of delivery of SCPs to PCPs Receipt and integration of SCPs by PCPs	Quantitative; cross-sectional	• Lack of technology to access SCPs on CD, unaware of secure fax • SCP associated with the date of cancer diagnosis and therefore behind other documents in EHR and difficult to locate	
Tevaarwerk et al. (2022)	Rural	*n* = 13 (clinicians) Not specified	EHR-based SCP template Assessed preferences for SCP elements	SCP information needs and preferences Usefulness of 16 SCP elements	Mixed; cross-sectional; interviews	• Primary care providers noted significant effort required to extract information from SCP to transfer to patient record • Lack of interoperability across EHR vendor systems • Lack of automatic pagination in the EHR to allow for the document to be faxable • Paper SCP	• Automated, integrated communication with updates pushed from oncology EHR to primary care EHR
Trosman et al. (2021)	Safety net hospitals (5/10 safety net institutions)	*n* = 888 (422 in the intervention cohort, 466 in the historical cohort) Breast	A structured care plan including cancer summary, care checklist, timing, and sequence of interdependent care	Usefulness of Care Sequence, a structured care plan including cancer summary, care checklist, timing, and sequence of interdependent care Patient self-management (know the stage of cancer, care plan clear or very clear, timing and sequence of care clear, seldom or never overwhelmed, not in control of care, able to manage and organize care well or very well, able to explain care to others well or very well, able to express preferences in care well or very well) Care delivery metrics (PCP consult, genetic consult, smoking cessation initiation, dental consult, fertility consult, flu vaccination)	Quantitative; cohort study		• Having key information on one sheet • Provision of structure to stay focused on the task • Ability to see a timeline and how long things will take • Proactively getting answers to questions • 47.2% preferred paper format • 40.3% preferred paper format with an electronic copy (6.6% preferred electronic copy)

#### Design-related factors

Lyson and colleagues found that none of the SCPs from 53 health systems caring for vulnerable populations was concordant with IOM recommendations for SCPs [50]. In addition, designing SCPs with input from diverse populations and tailoring SCPs for readability and cultural appropriateness could facilitate the implementation of SCPs [32, 45, 50]. However, cost in terms of staff time and resources posed a barrier to developing understandable and comprehensive SCPs [50, 51].

In 2 of the 19 studies, SCPs were re-engineered with input from healthcare providers caring for groups that were underserved. Among providers in a rural research network, design features that facilitated the use of SCPs included modifications like the addition of a date field to determine the outdatedness of contents, clearly labeling the cancer diagnosis as early as possible, focusing on follow-up care rather than treatment summaries, and removing screening recommendations that were not cancer-related [47]. Further, providers indicated a preference for having a single SCP for patients and themselves. In a study among providers, participants found SCPs to be too long [52]. Yet, they did not recommend the removal of any sections; instead, they recommended bulleted lists or summaries at the beginning of the SCP and suggested having separate SCPs for patients and providers [52].

Provider preferences for layout were predominantly based on a study of PCPs, who suggested labeling the sections on an electronic SCP, the addition of a date-created field for easier navigation, labeling cancer diagnosis clearly, direct links from treatment information to supporting documents [47], and a reminder function for appointments [32].

#### Organizational factors

Healthcare providers noted the lack of coordination between primary care and oncology as a barrier to the use of SCPs [25, 42, 53]. Primary care providers in a rural primary care practice reported either rarely or never receiving SCPs from oncology providers, did not have a formal process to identify cancer survivors, and were unaware of how to access resources for cancer survivors [53]. In a study based in a comprehensive cancer center treating poor, rural, and minority survivors, PCPs reported a low receipt of SCPs from oncologists sent via mail or fax [42]. Suggestions to facilitate the use of SCPs included one to two pages of specific recommendations from oncologists [53], periodic updates by oncologists [25], and health systems using a standard SCP to share with PCPs [51]. Isaacson and colleagues noted an organizational barrier in assessing the use of SCPs for survivors treated by providers, whose only affiliation with health systems may be for the purpose of using surgical facilities [51].

#### Technology-supported SCPs

Two studies focused on mHealth approaches to implementing SCPs. Based on an RCT of an SCP embedded in an mHealth app among racial and ethnic minorities, Psihogios and colleagues found that trivia questions and health goal messages increased engagement with the app, and this engagement varied by the season when messages were sent [54]. Baseman and colleagues explored the feasibility and acceptability of a mobile breast cancer survivorship care app among six rural breast cancer survivors, four PCPs, and one oncologist [32]. Survivors in this sample were enthusiastic about having one location for all information such as contact information, their treatment records, insurance numbers, and other relevant information. All survivors perceived their existing ways of self-management, surveillance, and monitoring as inadequate. The tool was seen as a better way to self-manage their survivorship and track symptoms, wellness activities, and mood. Portability was also seen as a plus as they always had their mobile phones available. Providers identified interoperability with other healthcare systems as a potential barrier, expressing concerns about data quality if the system relied on manual data entry [32].

#### Technology-related barriers

Isaacson and colleagues also highlighted implementation issues within two large rural health systems [51]. Operability was identified as a challenge within both institutions. Within one healthcare system, inefficiencies were identified within the ASCO template which could require multiple deletion of items unrelated to a patient’s treatment and survivorship. They used their Microsoft Word-friendly EHR system to prepare individualized SCPs. Whereas the other healthcare system integrated the SCP templates into their EHR that would populate the already existing data [51]. Multiple studies reported challenges with EHR integration [47, 52, 53], while Klemp et al. specifically reported difficulties in locating an SCP in the EHR [53].

## Discussion

The purpose of this study was to review the current evidence on the use of SCPs among groups that are underserved. We included 45 studies targeting various populations including racial and ethnic minorities (e.g., Blacks and Hispanics), low-income, and rural populations. Based on this review, we identified four overarching themes with respect to the implementation of SCPs among these populations. First, groups that are underserved report the benefits of using SCPs. Second, despite acknowledging benefits, these groups also report lower rates of receiving SCPs compared to their non-Hispanic White counterparts. Third, there are substantial unmet information needs, as well as preferences, within this population that are not being addressed. Fourth, tailoring SCPs to target populations, addressing organizational barriers to disseminating SCPs, and electronic mode of delivery may improve knowledge retention and acceptability of these tools among survivors and their providers.

### Benefits of SCPs exist among underserved populations

Our review found several benefits of SCPs, including improved care coordination (e.g., timely follow and adherence to care) and self-management of cancer. These findings align with those of Lewis-Thames and colleagues, who found benefits such as improving disease management and aiding patient-provider communication and care coordination among minority breast cancer survivors [19]. In addition, reviews among the general population of cancer survivors also found positive findings for proximal outcomes such as adherence to care for patients [16] and care coordination for providers [16, 55]. However, much of the attention in the field has focused on the mixed evidence associated with the impact of SCPs on more distal outcomes such as health outcomes [16, 17]. However, it should be noted that underserved populations may be underrepresented in this research. When populations are pooled together, interesting and unique differences may be washed out. In addition, underserved populations may benefit more from improvements in these proximal outcomes which are contributors to health disparities which could ultimately lead to improvements in health outcomes and health equity among these populations.

### Disparities in receipt of SCPs further limit their benefits

While there are potential benefits to the use of SCPs among underserved populations, these benefits cannot be fully realized due to disparities in the receipt of SCPs. Our study found that groups that are underserved reported lower rates of receiving treatment summaries, follow-up care instructions, and survivorship self-care. Previous studies have also reported on the lack of adoption of SCPs [56, 57]. Several barriers exist when it comes to adopting and implementing SCPs including the lack of reimbursement for preparing SCPs, as well as the lack of time and resources. These barriers may be more pronounced among providers predominantly serving racial and ethnic minority populations, rural populations, and low-income populations, leading to lower adoption rates among these populations. In addition, not all professional societies require the use of SCPs for cancer care providers to receive accreditation. This makes the adoption and use of SCPs discretionary [57]. A recent study conducted in Maryland has also reported that Black female cancer survivors are more likely to receive SCPs compared to non-Hispanic White cancer survivors [58] which could be a result of these patients having providers who are more likely to provide their patients with SCPs.

### A case for risk-stratified SCPs

The IOM highlighted the need to recognize survivorship as a distinct phase in cancer care and the need to address the specific concerns of survivors [10]. Survivorship care plans serve as a roadmap for patients and as a communication tool between cancer care providers and PCPs [59]. However, enthusiasm for using SCPs has been tempered by the evidence that SCPs are not effective and are exceedingly difficult to effectively implement [16, 17]. While SCPs are still recommended for cancer patients, SCPs are no longer mandated by accreditation bodies including the Commission on Cancer (COC) [60]. Per the 2020 COC guidelines, particular elements of SCPs are not mandated [61]. Importantly, they also recommend that cancer programs define the population to receive SCPs [61].

Recent attention has been given to risk-stratified survivorship care models. Using this model, survivorship care is tailored based on risk. For example, survivors with low-risk for late effects resulting from treatment may be transitioned to receive care from PCPs, whereas high-risk groups would continue to receive more specialized care from their oncology team to manage their complex condition [62, 63]. In addition to benefiting patients, this model also benefits providers by reserving the specialty oncology clinics for patients in greater need, and not burdening PCPs with complex patients [64]. In this case, socially vulnerable survivors would be identified as high risk for poor cancer outcomes. These populations would benefit from cancer programs devoting resources to the delivery of SCPs which would also improve proximal outcomes such as adherence to follow-up and cancer coordination which could lead to improvements in distal outcomes and reductions in cancer disparities.

### Needs and preferences differ by underserved group

Importantly, the provision of SCPs alone is not enough to see these potential benefits. SCPs must be implemented to meet the needs and preferences of populations of interest. This study also found that groups that are underserved have substantial unmet needs with respect to their survivorship care. Importantly, among those who do receive some form of survivorship care planning, these populations tend to report that SCPs do not meet their current needs. Similarly, other studies have found greater informational and unmet needs among African-Americans [65], other minorities [66], and low-income populations. It is also reported that differences in information needs among minorities and non-Hispanic Whites exist after controlling for income status [67]. Cancer survivors with unmet information needs are more likely to experience psychological distress and lower perceptions of health competence and well-being. Providing this population with adequate information in the form of SCPs can improve health-related quality of life and psychosocial health and reduce depression [67].

In addition, we found that the needs and preferences differ by respective population. Addressing the specific needs and preferences of a population may call for alternative methods and timing of delivery of post-treatment education and care planning. For example, Hispanic cancer survivors reported information needs in areas related to finances, continuity of care, and symptom management. Important sources of additional support for this population are patient navigators [48].

### The importance of tailoring SCPs

Groups that are underserved would benefit from tailored SCPs to facilitate optimal survivorship care. This is in alignment with several studies that have identified a need for survivorship planning that is robust and individualized based on the unique needs of the population [68–70]. Moreover, a previous review looking at survivors’ experiences using SCPs highlighted the importance of individualized SCPs that reflect the key concerns of cancer survivors [71]. Studies also report that while cancer survivors are receptive to SCPs, they often view them as too technical [45]. It has been established that cancer survivors are frequently excluded from the development process of SCPs, resulting in care plans not being targeted to their specific needs and preferences [72].

### Barriers and challenges to consider

Our study also found several barriers to the implementation of SCPs. These included costs associated with the development of SCPs such as staff time and resources, as well as a lack of coordination between primary care and oncology with respect to dissemination. Previous reviews have also found a common barrier in SCPs often not being delivered to survivors or PCPs, partly due to EHR capabilities and interoperability between systems [57]. The literature also supports the lack of organizational resources being a barrier to SCP use [17], even when mandated by professional societies. As mentioned previously, SCPs are no longer mandated by COC [60]. Importantly, the provision of SCPs to underserved populations would also require the devotion of additional resources to the implementation of SCPs. This may be burdensome for institutions whose resources are severely limited, especially minority-serving and safety-net cancer centers. In addition, professional societies may also be able to offer assistance and provide information to these institutions which highlight barriers and offer methods to facilitate their implementation and use.

### Gaps in the literature

Our findings also highlight the knowledge gaps to direct future research efforts. First, oncologist perspectives were underrepresented in our review. Second, while individual-level sociodemographic factors have been assessed for their association with the receipt of SCPs, organizational factors influencing the implementation of SCPs have been understudied. Third, while studies recognize the need for culturally tailored SCPs, there were few instances of their implementation and evaluation. Lastly, few studies focused on the benefits of SCPs among groups that are underserved. For example, future research could explore the relationship between the receipt of SCPs and how it influences outcomes such as the receipt of guideline-concordant treatment and follow-up care.

### Strengths and limitations

The main strength of this review is that it provides a systematic synthesis of the scientific evidence on the use of SCPs among groups that are underserved. While current evidence provides little support for the role of SCPs in improving health outcomes and healthcare delivery, there is evidence that it can improve the coordination of care. SCPs also serve as a communication tool between oncologists and PCPs [59]. Having these tools available may lead to patient activation among these cancer survivors, empowering them to manage, coordinate, and advocate for their survivorship care [73]. Improving care coordination and reducing barriers to care may improve health disparities and clinical outcomes among groups that are underserved.

This study has several limitations. One potential limitation is that our search strategy may not have captured all potential articles meeting our inclusion criteria. Due to variations in the definitions of SCPs, some articles may not have been included. However, we performed a snowball review of the references for included studies to minimize this. In addition, another limitation is due to the heterogeneity in study design and types of outcomes evaluated, we were unable to aggregate findings in the manner of a meta-analysis. However, the insights gleaned from the qualitative studies incorporated in our review offer valuable perspectives and enrich the depth of our findings. Our review also synthesized findings from articles of adolescent and young adult childhood cancer survivors, where it was not possible to provide the exact numbers of non-adult participants. Similarly, we included articles based on secondary analyses of data, where nearly half the sample was composed of low SES groups, but these studies did not report results specifically for these groups. Finally, the included papers may be subject to publication bias as studies that report negative findings are less likely to be published.

## Conclusion

Overall, this scoping review on the use of SCPs among cancer survivors from groups that are underserved found that there were disparities in the receipt of SCPs and that the content of existing SCPs does not align with survivor needs and preferences. SCPs tailored to the specific needs of these populations in terms of content, mode, and timing of delivery are likely to improve acceptability of SCPs and facilitate better cancer care delivery.

## Supplementary Information

Below is the link to the electronic supplementary material.

Supplementary file1 (DOCX 13 KB)

Supplementary file2 (DOCX 27.2 KB)

## Data Availability

All relevant data are within the manuscript and its Supplementary Information files.
