# Supplementary material for: A scoping review of the evidence on survivorship care plans among minority, rural, and low-income populations
Source: J Cancer Surviv. 2024 Jun 22;19(6):1956–94. doi: 10.1007/s11764-024-01609-z (PMC12546521; doi:10.1007/s11764-024-01609-z)
Supplement: Supplementary file 1 — Supplementary file1 (DOCX 13 KB) [file 11764_2024_1609_MOESM1_ESM.docx]

**Appendix 1. Operationalization of the search terms.**

| Category | Search terms |
| --- | --- |
| Cancer | Cancer, neoplasms |
| Survivorship Care Plans | Patient care planning, care plan/planning, follow-up care |
| Underserved populations | Ethnic, race, racial, disparity, minority, underserved, rural, elderly, low-income, poverty, hispanic, mexican, latino, african, black, Asian, american indian, alaskan native, native american, inuit or pacific islander |

Note: Search terms within each category are combined with the OR operator. Search terms between categories are combined with the AND operator. Some terms were truncated to capture keywords with the same stem.
