# Supplementary material for: A scoping review of the evidence on survivorship care plans among minority, rural, and low-income populations
Source: J Cancer Surviv. 2024 Jun 22;19(6):1956–94. doi: 10.1007/s11764-024-01609-z (PMC12546521; doi:10.1007/s11764-024-01609-z)
Supplement: Supplementary file 2 — Supplementary file2 (DOCX 27.2 KB) [file 11764_2024_1609_MOESM2_ESM.docx]

**Appendix 2: Included Studies (n=45)**

Alford-Teaster, J., Vaclavik, D., Imset, I., Schiffelbein, J., Lyons, K., Kapadia, N., Olson, A., McGrath, E. B., Schifferdecker, K., & Onega, T. (2023). From active treatment to surveillance: How the barriers and facilitators of implementing survivorship care planning could be an opportunity for telehealth in oncology care for rural patients. *Journal of Cancer Survivorship*. <https://doi.org/10.1007/s11764-023-01447-5>

Arana-Chicas, E., Prisco, L. M. H., Sharma, S., Stauffer, F., McGee, M., Dauphin, S., Ban-Hoefen, M., Navarette, J., Zittel, J., Cupertino, A. P., Magnuson, A., Mustian, K. M., & Mohile, S. G. (2023). Cancer survivorship challenges of rural older adults: A qualitative study. *BMC Cancer*, *23*(1), 917. <https://doi.org/10.1186/s12885-023-11395-z>

Ashing, K., Serrano, M., Weitzel, J., Lai, L., Paz, B., & Vargas, R. (2014). Towards developing a bilingual treatment summary and survivorship care plan responsive to Spanish language preferred breast cancer survivors. *Journal of Cancer Survivorship*, *8*(4), 580-594. <https://doi.org/10.1007/s11764-014-0363-5>

Ashing-Giwa, K., Tapp, C., Brown, S., Fulcher, G., Smith, J., Mitchell, E., Santifer, R. H., McDowell, K., Martin, V., Betts-Turner, B., Carter, D., Rosales, M., & Jackson, P. A. (2013). Are survivorship care plans responsive to African-American breast cancer survivors? Voices of survivors and advocates. *Journal of Cancer Survivorship*, *7*(3), 283-291. <https://doi.org/10.1007/s11764-013-0270-1>

Baseman, J., Revere, D., & Baldwin, L. M. (2017). A mobile breast cancer survivorship care app: Pilot study. *JMIR Cancer*, *3*(2), e14. <https://doi.org/10.2196/cancer.8192>

Burg, M. A., Lopez, E. D., Dailey, A., Keller, M. E., & Prendergast, B. (2009). The potential of survivorship care plans in primary care follow-up of minority breast cancer patients. *Journal of General Internal Medicine*, *24 Suppl 2*(Suppl 2), S467-471. <https://doi.org/10.1007/s11606-009-1012-y>

Burke, N. J., Napoles, T. M., Banks, P. J., Orenstein, F. S., Luce, J. A., & Joseph, G. (2016). Survivorship care plan information needs: Perspectives of safety-net breast cancer patients. *PloS One*, *11*(12), e0168383. <https://doi.org/10.1371/journal.pone.0168383>

Casillas, J., Syrjala, K. L., Ganz, P. A., Hammond, E., Marcus, A. C., Moss, K. M., Crespi, C. M., Lu, P., McCabe, M. S., Ford, J. S., Jacobs, L. A., Pucci, D., Palmer, S. C., Termuhlen, A. M., Diller, L., Campbell, M., Jones, B., & Friedman, D. L. (2011). How confident are young adult cancer survivors in managing their survivorship care? A report from the LIVESTRONG™ Survivorship Center of Excellence Network. *Journal of Cancer Survivorship*, *5*(4), 371-381. <https://doi.org/10.1007/s11764-011-0199-1>

Casillas, J. N., Bolano, C., Schwartz, L. F., Ganz, P. A., Kahn, K., Stuber, M., Bastani, R., Morales, S., Macadangdang, J., Lidington, E. K., Quintana, K., Gonzalez, A., Casas, E., & Barboa, E. (2022). A survivorship educational tool for Latino adolescent and young adult cancer survivors. *Health Promot Pract*, *23*(5), 861-873. <https://doi.org/10.1177/15248399211061709>

Casillas, J. N., Schwartz, L. F., Gildner, J. L., Crespi, C. M., Ganz, P. A., Kahn, K. L., Stuber, M. L., Bastani, R., Morales, S., Quintana, K., Gonzalez, A., Casas, E., & Barboa, E. (2021). Engaging Latino adolescent and young adult (AYA) cancer survivors in their care: Piloting a photonovela intervention. *Journal of Cancer Education*, *36*(5), 971-980. <https://doi.org/10.1007/s13187-020-01724-2>

DeGuzman, P., Colliton, K., Nail, C. J., & Keim-Malpass, J. (2017). Survivorship care plans: Rural, low-income breast cancer survivor perspectives. *Clinical Journal of Oncology Nursing*, *21*(6), 692-698. <https://doi.org/10.1188/17.Cjon.692-698>

Desmond, R. A., Jackson, B. E., & Waterbor, J. W. (2017). Disparities in cancer survivorship indicators in the deep south based on BRFSS data: Recommendations for survivorship care plans. *Southern Medical Journal*, *110*(3), 181-187. <https://doi.org/10.14423/smj.0000000000000617>

Duggan, C., Cushing-Haugen, K. L., Cole, A. M., Allen, J., Gilles, R., Hornecker, J. R., Gutierrez, A. I., Warner, J., Baker, K. S., Ceballos, R. M., & Chow, E. J. (2023). Feasibility of delivering survivorship care via lay health educators: A pilot randomized controlled trial among rural cancer survivors. *Journal of Rural Health*, *39*(3), 666-675. <https://doi.org/10.1111/jrh.12736>

Greenlee, H., Molmenti, C. L., Crew, K. D., Awad, D., Kalinsky, K., Brafman, L., Fuentes, D., Shi, Z., Tsai, W. Y., Neugut, A. I., & Hershman, D. L. (2016). Survivorship care plans and adherence to lifestyle recommendations among breast cancer survivors. *Journal of Cancer Survivorship*, *10*(6), 956-963. <https://doi.org/10.1007/s11764-016-0541-8>

Hershman, D. L., Greenlee, H., Awad, D., Kalinsky, K., Maurer, M., Kranwinkel, G., Brafman, L., Jayasena, R., Tsai, W. Y., Neugut, A. I., & Crew, K. D. (2013). Randomized controlled trial of a clinic-based survivorship intervention following adjuvant therapy in breast cancer survivors. *Breast Cancer Research and Treatment*, *138*(3), 795-806. <https://doi.org/10.1007/s10549-013-2486-1>

Hinyard, L., & Wirth, L. S. (2017). Race is a strong predictor of receipt of a written survivorship care plan: Results from the National Health Interview Survey. *Journal of Community Health*, *42*(6), 1156-1162. <https://doi.org/10.1007/s10900-017-0365-0>

Isaacson, M. J., Hulme, P. A., Cowan, J., & Kerkvliet, J. (2018). Cancer survivorship care plans: Processes, effective strategies, and challenges in a Northern Plains rural state. *Public Health Nursing*, *35*(4), 291-298. <https://doi.org/10.1111/phn.12393>

Jabson, J. M., & Bowen, D. J. (2013). Cancer treatment summaries and follow-up care instructions: Which cancer survivors receive them? *Cancer Causes and Control*, *24*(5), 861-871. <https://doi.org/10.1007/s10552-013-0163-7>

Kantsiper, M., McDonald, E. L., Geller, G., Shockney, L., Snyder, C., & Wolff, A. C. (2009). Transitioning to breast cancer survivorship: Perspectives of patients, cancer specialists, and primary care providers. *Journal of General Internal Medicine*, *24 Suppl 2*(Suppl 2), S459-466. <https://doi.org/10.1007/s11606-009-1000-2>

Kenzik, K. M., Kvale, E. A., Rocque, G. B., Demark-Wahnefried, W., Martin, M. Y., Jackson, B. E., Meneses, K., Partridge, E. E., & Pisu, M. (2016). Treatment summaries and follow-Up care instructions for cancer survivors: Improving survivor self-efficacy and health care utilization. *Oncologist*, *21*(7), 817-824. <https://doi.org/10.1634/theoncologist.2015-0517>

Kim, K., Xu, W., Hong, S. J., Starkweather, A., Brown, R. F., & Walsh, S. (2022). Perceived discrimination and physical activity mediate the associations between receiving a survivorship care plan and cancer pain. *Cancer Epidemiology*, *78*, 102155. <https://doi.org/10.1016/j.canep.2022.102155>

Klemp, J. R., Knight, C. J., Lowry, B., Long, T., Bush, C., Alsman, K., Krebill, H., Peereboom, D., Overholser, L., & Greiner, K. A. (2022). Informing the delivery of cancer survivorship care in rural primary care practice. *Journal of Cancer Survivorship*, *16*(1), 4-12. <https://doi.org/10.1007/s11764-021-01134-3>

Ko, E., Cardenas, V., Zúñiga, M. L., Woodruff, S. I., Rodriguez, V., & Palomino, H. (2021). Challenges for Latina breast cancer patient survivorship care in a rural US-Mexico border region. *International Journal of Environmental Research and Public Health*, *18*(13). <https://doi.org/10.3390/ijerph18137024>

Ko, E., Woodruff, S. I., Zúñiga, M. L., Cardenas, V., Lizarraga, M., & Urias, A. (2023). Culturally-tailored survivorship care planning for rural Latina breast cancer patients: A pilot study. *Journal of Psychosocial Oncology*, *41*(4), 475-486. <https://doi.org/10.1080/07347332.2022.2141168>

Ko, E., Zúñiga, M. L., Woodruff, S. I., Serra-Martinez, Y., & Cardenas, V. (2020). Development of a survivorship care plan (SCP) program for rural Latina breast cancer patients: Proyecto Mariposa-application of intervention mapping. *International Journal of Environmental Research and Public Health*, *17*(16). <https://doi.org/10.3390/ijerph17165784>

Lewis-Thames, M. W., Carnahan, L. R., James, A. S., Watson, K. S., & Molina, Y. (2020). Understanding posttreatment patient-provider communication and follow-up care among self-identified rural cancer survivors in Illinois. *Journal of Rural Health*, *36*(4), 549-563. <https://doi.org/10.1111/jrh.12414>

Linscott, J. A., Rutan, M. C., Han, P. K. J., Hansen, M. H., Hayn, M. H., Ryan, S. T., Trinh, Q. D., & Sammon, J. D. (2020). Receipt of survivorship care plans and self-reported health status among patients with genitourinary malignancy. *Journal of Urology*, *204*(3), 564-569. <https://doi.org/10.1097/ju.0000000000001032>

Lyson, H. C., Haggstrom, D., Bentz, M., Obeng-Gyasi, S., Dixit, N., & Sarkar, U. (2021). Communicating critical information to cancer survivors: An assessment of survivorship care plans in use in diverse healthcare settings. *Journal of Cancer Education*, *36*(5), 981-989. <https://doi.org/10.1007/s13187-020-01725-1>

Malhotra, J., Paddock, L. E., Lin, Y., Pine, S. R., Habib, M. H., Stroup, A., & Manne, S. (2023). Racial disparities in follow-up care of early-stage lung cancer survivors. *Journal of Cancer Survivorship*, *17*(5), 1259-1265. <https://doi.org/10.1007/s11764-022-01184-1>

Maly, R. C., Liang, L. J., Liu, Y., Griggs, J. J., & Ganz, P. A. (2017). Randomized controlled trial of survivorship care plans among low-income, predominantly Latina breast cancer survivors. *Journal of Clinical Oncology*, *35*(16), 1814-1821. <https://doi.org/10.1200/jco.2016.68.9497>

Millar, M. M., Herget, K. A., Ofori-Atta, B., Codden, R. R., Edwards, S. L., Carter, M. E., Belnap, B., Kirchhoff, A. C., & Sweeney, C. (2023). Cancer survivorship experiences in Utah: An evaluation assessing indicators of survivors' quality of life, health behaviors, and access to health services. *Cancer Causes and Control*, *34*(4), 337-347. <https://doi.org/10.1007/s10552-023-01671-5>

Nápoles, A. M., Santoyo-Olsson, J., Chacón, L., Stewart, A. L., Dixit, N., & Ortiz, C. (2019). Feasibility of a mobile phone app and telephone coaching survivorship care planning program among Spanish-speaking breast cancer survivors. *JMIR Cancer*, *5*(2), e13543. <https://doi.org/10.2196/13543>

Psihogios, A. M., King-Dowling, S., O'Hagan, B., Darabos, K., Maurer, L., Young, J., Fleisher, L., Barakat, L. P., Szalda, D., Hill-Kayser, C. E., & Schwartz, L. A. (2021). Contextual predictors of engagement in a tailored mHealth intervention for adolescent and young adult cancer survivors. *Annals of Behavioral Medicine*, *55*(12), 1220-1230. <https://doi.org/10.1093/abm/kaab008>

Rencsok, E. M., Stopsack, K. H., Slopen, N., Odedina, F. T., Ragin, C., Nowak, J., McSwain, L., Manarite, J., Heath, E., George, D. J., Kantoff, P. W., Vinson, J., Villanti, P., Haneuse, S., & Mucci, L. A. (2023). Experience with the US health care system for Black and White patients with advanced prostate cancer. *Cancer*, *129*(16), 2532-2541. <https://doi.org/10.1002/cncr.34885>

Rutledge, T. L., Kano, M., Guest, D., Sussman, A., & Kinney, A. Y. (2017). Optimizing endometrial cancer follow-up and survivorship care for rural and other underserved women: Patient and provider perspectives. *Gynecologic Oncology*, *145*(2), 334-339. <https://doi.org/10.1016/j.ygyno.2017.03.009>

Sabatino, S. A., Thompson, T. D., Smith, J. L., Rowland, J. H., Forsythe, L. P., Pollack, L., & Hawkins, N. A. (2013). Receipt of cancer treatment summaries and follow-up instructions among adult cancer survivors: Results from a national survey. *Journal of Cancer Survivorship*, *7*(1), 32-43. <https://doi.org/10.1007/s11764-012-0242-x>

Shay, L. A., Schmidt, S., Dioun, S. I., Grimes, A., & Embry, L. (2019). Receipt of a survivorship care plan and self-reported health behaviors among cancer survivors. *Journal of Cancer Survivorship*, *13*(2), 180-186. <https://doi.org/10.1007/s11764-019-00740-6>

Stewart, T. P., Sesto, M. E., Haine, J. E., Henningfield, M. F., Norslien, K., Zhang, X., Hahn, D. L., & Tevaarwerk, A. J. (2022). Results of engineering, primary care, oncology collaborative regarding a survey of primary care on a re-engineered survivorship care plan. *Journal of Cancer Education*, *37*(1), 23-29. <https://doi.org/10.1007/s13187-020-01776-4>

Tawfik, B., Jaffe, S. A., Mohler, L., Oomen-Hajagos, J., Gil, I. S., Chamberlain, R., Gagnon, S., Kano, M., Gundelach, A., Ryan, S. R., Abernathy, J., Wiggins, C., Sussman, A., & Dayao, Z. (2021). Developing a survivorship care plan (SCP) delivery process for patients and primary care providers serving poor, rural, and minority patients with cancer. *Supportive Care in Cancer*, *29*(9), 5021-5028. <https://doi.org/10.1007/s00520-021-06043-w>

Tevaarwerk, A. J., Zhang, X., Haine, J., Norslien, K., Henningfield, M. F., Stietz, C., Hahn, D., & Sesto, M. E. (2022). Re-engineering survivorship care plans to support primary care needs and workflow: Results from an Engineering, Primary care and Oncology Collaborative for Survivorship Health (EPOCH). *Journal of Cancer Education*, *37*(6), 1654-1661. <https://doi.org/10.1007/s13187-021-02008-z>

Timsina, L. R., Zarzaur, B., Haggstrom, D. A., Jenkins, P. C., Lustberg, M., & Obeng-Gyasi, S. (2021). Dissemination of cancer survivorship care plans: Who is being left out? *Supportive Care in Cancer*, *29*(8), 4295-4302. <https://doi.org/10.1007/s00520-020-05915-x>

Tisnado, D. M., Mendez-Luck, C., Metz, J., Peirce, K., & Montaño, B. (2017). Perceptions of survivorship care among Latina women with breast cancer in Los Angeles county. *Public Health Nursing*, *34*(2), 118-129. <https://doi.org/10.1111/phn.12299>

Trosman, J. R., Weldon, C. B., Rapkin, B. D., Benson, A. B., 3rd, Makower, D. F., Liang, S. Y., Kulkarni, S. A., Perez, C. B., Lo, S. S., Krueger, E. A., Throckmorton, A. D., Gallagher, C., Hoskins, K., Schaeffer, C. M., Van Horn, J., Schapira, L., Ravelo, A., Yu, E., & Gradishar, W. J. (2021). Evaluation of the Novel 4R oncology care planning model in breast cancer: Impact on patient self-management and care delivery in safety-net and non-safety-net centers. *JCO Oncol Pract*, *17*(8), e1202-e1214. <https://doi.org/10.1200/op.21.00161>

Wen, K. Y., Hu, A., Ma, G. X., Fang, C. Y., & Daly, M. B. (2014). Information and communication needs of Chinese American breast cancer patients: Perspectives on survivorship care planning. *J Community Support Oncol*, *12*(12), 439-445. <https://doi.org/10.12788/jcso.0095>

Wu, J., Blair, J., Izevbigie, O. C., Wright, N. C., & Arend, R. C. (2018). Disparities in receipt of follow-up care instructions among female adult cancer survivors: Results from a national survey. *Gynecologic Oncology*, *150*(3), 494-500. <https://doi.org/10.1016/j.ygyno.2018.06.024>
